# Reliable Time Propagation Algorithms for PMF and RBPMF

**DOI:** 10.3390/s21010261

**Published:** 2021-01-02

**Authors:** Chang-Ky Sung, Sang Jeong Lee

**Affiliations:** 1 Agency for Defense Development, Yuseong P.O. Box 35, Daejeon 34186, Korea; 2 Department of Electronics Engineering, Chungnam National University, 99 Daehak-ro, Yuseong-gu, Daejeon 34134, Korea; eesjl@cnu.ac.kr

**Keywords:** point mass filter, Rao–Blackwellized point mass filter, mass redefinition, Moment Matched Gaussian Kernel

## Abstract

This paper addresses the reliable time propagation algorithms for Point Mass Filter (PMF) and Rao–Blackwellized PMF (RBPMF) for the nonlinear estimaton problem. The conventional PMF and RBPMF process the probability diffusion for the time propagation with the direct sampled-values of the process noise. However, if the grid interval is not dense enough, it fails to represent the statistical characteristics of the noise accurately so the performance might deteriorate. To overcome that problem, we propose time propagation convolution algorithms adopting Moment Matched Gaussian Kernel (MMGK) on regular grids through mass linear interpolation. To extend the dimension of the MMGK that can accurately describe the noise moments up to the kernel length, we propose the extended MMGK based on the outer tensor product. The proposed time propagation algorithms using one common kernel through the mass linear interpolation not only improve the performance of the filter but also significantly reduce the computational load. The performance improvement and the computational load reduction of the proposed algorithms are verified through numerical simulations for various nonlinear models.

## 1. Introduction

Recursive Bayesian filtering recursively predicts and corrects an unknown Probability Density Function (PDF) using a mathematical model and incoming measurements. However, it is almost impossible to obtain a closed-form solution for all estimation problems by applying Bayesian filtering technique. As a very limited case, if the model is linear and all the random variables included in the model follow normal distributions, there exists an explict solution for Bayesian filtering, and that solution is well-known Kalman Filter (KF) [1,2]. For other general problems, two groups of techniques are applied to obtain approximate solutions.

The first group is based on the assumption that, despite the nonlinearity of the model, PDFs to be estimated follow normal distributions approximately. Among them, the most representative nonlinear filter widely applied in various fields is Extended Kalman Filter (EKF) [1,2], which applies KF after linearizing the model. EKF has the advantage of showing excellent asymptotic convergence characteristics despite linearization errors [3,4]. Methods which do not apply model linearization include Unscented Kalman Filter (UKF) and Ensemble Kalman Filter (EnKF) which approximate PDFs to normal distributions [5,6]. Gaussian Filters (GFs) apply Gaussian quadrature integration rules to calculate expectations of nonlinear functions for normal distributions [7].

The second group directly estimate PDFs by applying numerical approximation techniques to solve Bayesian filtering. Particle Filter (PF) is the most representative method. The concept of PF was first proposed in the 1950s, but it was not popular until Gordon proposed the bootstrap algorithm that the PF established itself as a representative nonlinear-/non-gaussian estimation technique with high accuracy [1,2,5,7,8,9]. PF directly estimates non-gaussian PDFs through the combination of positions and weights of random sampling points. It is known that the estimation error of PF is independent of the dimension of the state variable [10,11]. In the practical application of PF, the increase of the computational load with dimension is still a problem to be tackled [2,9]. Rao–Blackwellized PF (RBPF), which divides the state variable into a nonlinear part and a linear part, and applies PF to the nonlinear part and KF to the linear part, is a state-of-the-art computational load reduction technique [12,13].

PMF is another method obtaining an approximate Bayesian filtering solution [2,5,14]. PMF estimates PDFs using masses of equally spaced grids on a state space. PMF was introduced in the early 1970s because of its conceptual simplicity [15], and the advantage of the PMF over the PF is its deterministic nature of the algorithm. However, it has not received as much attention as the PF due to the lack of efficient grid design methods or a problem of excessive computation. However, developments of computing technologies led to the introduction of a PMF-based TRN algorithm in the 1990s. Since then, there have been active researches for performance improvements of PMF [16,17]. Most of the studies have been conducted for the purpose of improving TRN performance, but the results are applicable to general estimation problems. Numerous research results for efficient grid design or reselection have been presented to improve PMF performance, and there are the Anticipative Grid Design (AGD) algorithm and the Boundary-based Grid Design (BGD) algorithm [18], the grid resolution and support design algorithm considering a noise level in TRN [19], the grid support design algorithm using mutual information [20], the density specific grid design algorithm assuming two different grids [21], and the density difference grid design algorithm based on the differentiation of the PDF in a sparse grid [22].

In implementing the filter, it is important to process probability diffusion through time propagation for the filter performance. In the case of PMF, time propagation is conceptually a convolution operation between masses of grids and the kernel of the process noise. The kernel is a set of the directly sampled values of the process noise PDF with arguments of differences between irregular grids passing a system model and newly defined regular grids. However, if the grid interval is not dense enough, the conventional kernel cannot accurately represent the statistical characteristics of the noise. This causes a problem in which the probability diffusion is not properly handled so the filter performance might deteriorate [23]. To resolve this problem, Variance Adjusted Gaussian Kernel (VAGK) or Moment Matched Gaussain Kernel (MMGK) have been proposed [23,24]. As another countermeasure, the Density-Weighted Convolution (DWC) algorithm, dealing with a model that only the measurement model is nonlinear, has been proposed recently [25]. Among them, MMGK exactly matches the moments of the noise up to the effective kernel length. However, those are kernel generation techniques applicable only for the model whose system model is linear. That is, it cannot be directly applied to irregular grid intervals due to the nonlinearity of the system model. In this paper, as a first result, we propose the PMF algorithm with indirect time propagation using MMGK through a mass redefinition process for general nonlinear estimation problems. The proposed algorithm has the advantage of not only improving the performance but also reducing the burden of calculating the direct-sampled values of the process noise. Furthermore, we propose the dimension extended MMGK by applying outer tensor product.

Like PF, PMF cannot be free from the problem of the extensive computational burden. In particular, PMF is limited to low-dimensional problems due to the high-dimensional convolution operation. In order to reduce the computational load of PMF, RBPMF, analogous to RBPF, has been proposed relatively recently [26]. RBPMF algorithm for easy implementation of measurement validity check logic, which is essential for maintaining the filter stability in practical application, and RBPMF for TRN estimation problems were proposed [27,28]. Recently, Rao–Blackwellized Particle-Point Mass Fusion Filter (RBPPFF), which combines RBPF and RBPMF, has been introduced for robust TRN [29]. However, the proposed algorithms have the same abnormal probability diffusion problem in the time propagation operation as the aforementioned PMF. In this paper, as a second result, we propose the RBPMF algorithm applying MMGK without a mass redefinition.

In RBPMF, the weight of each grid for the nonlinear part is paired with the normal distribution for the linear part. Since the linear part acts as an artifact noise to the nonlinear part, it is impossible to apply a common kernel to the time propagation of the nonlinear part. However, if model terms related to the linear part are not functions of the nonlinear part like TRN, all covariances of the linear part approximately have the same value. So, for these specific models, we can apply the time propagation operation to RBPMF through grid and mass redefinition, like the proposed PMF. To complete this scheme, a linear part redefinition procedure following the nonlinear part mass redefinition is required. However, until now, only the index-based adaption algorithm of simply copying the state of the neighboring linear part for the TRN problem has been proposed [30]. Therefore, for the constant linear model case, we propose the RBPMF algorithm with indirect time propagation, which includes the redefinition process of the linear part, as the third result of this paper.

The composition of this paper is as follows. First, we introduce Bayesian filtering in Section 2, then describe the conventional PMF and the proposed PMF algorithm with the indirect time propagation algorithm, and then show the simulation results comparing their performances. Section 4 describes the conventional RBPMF and two proposed RBPMF algorithms, simulation results for them, and concludes in Section 5.

## 2. Bayesian Filtering

Let us consider the following nonlinear discrete-time stochastic dynamic system.

(1) $$\begin{array}{ccc} x_{k} & = & f_{k-1}\left(x_{k-1}\right)+w_{k-1} \end{array}$$

(2) $$\begin{array}{ccc} y_{k} & = & h_{k}\left(x_{k}\right)+v_{k} \end{array}$$

where $x\in R^{n}$ is the state variable to be estimated and $y_{k}\in R^{m}$ is the measurement. It’s assumed that the process noise $w_{k}$ and the measurement noise $v_{k}$ are white noise and mutually independent of each other, and follow known normal distributions, $p_{w_{k}}\left(w_{k}\right)$ and $p_{v_{k}}\left(v_{k}\right)$, respectively. The nonlinear mappings $f_{k}:R^{n}\to R^{n}$ and $h_{k}:R^{n}\to R^{m}$ represent the system model and the measurement model. The model in Equation (1) is an input-free model, but can be easily extended to an input-driven one.

The conditional PDFs of the state variable given measurements, which is the estimation target of Bayesian filtering, is $p(x_{k}|Y_{k})$. Here, $Y_{k}=\left\{y_{0},y_{1},\cdots ,y_{k}\right\}$ is a set of all measurements up to time $t_{k}$. Two PDFs to be estimated are the priori PDF $p(x_{k}|Y_{k-1})$ which is one step ahead prediction and the posterori PDF $p(x_{k}|Y_{k})$ which is filtering.

To solve a recursive Bayesian filtering problem, PDF models are required. First, the transition PDF of the state variable for the system model in Equation (1) is as follows.

(3) $$x_{k}∼p\left(x_{k}|x_{k-1}\right)=p_{w_{k-1}}\left(x_{k}-f_{k-1}\left(x_{k-1}\right)\right)$$

The transition PDF satisfies $p\left(x_{k}|x_{k-1},Y_{k-1}\right)=p\left(x_{k}|x_{k-1}\right)$ from the Markovian characteristic. The PDF model for the measurement equation is as follows.

(4) $$y_{k}∼p\left(y_{k}|x_{k}\right)=p_{v_{k}}\left(y_{k}-h_{k}\left(x_{k}\right)\right)$$

$p\left(y_{k}|x_{k},Y_{k-1}\right)=p\left(y_{k}|x_{k}\right)$ is satisfied because $y_{k}$ is dependent only on $x_{k}$ from the measurement model.

Bayesian filtering is a process of recursively obtaining the posteriori PDF $p(x_{k}|Y_{k})$ for the current time under the assumption that the posteriori PDF $p(x_{k-1}|Y_{k-1})$ for the previous time is known. To solve this, first, apply Bayes’ theorem to the posteriori PDF, then $p(x_{k}|Y_{k})$ becomes as in Equation (5).

(5) $$p\left(x_{k}|Y_{k}\right)=\frac{p\left(y_{k}|x_{k},Y_{k-1}\right)p\left(x_{k}|Y_{k-1}\right)}{p(y_{k}|Y_{k-1})}$$

If $p\left(y_{k}|x_{k},Y_{k-1}\right)=p\left(y_{k}|x_{k}\right)$ is applied to Equation (5), $p(x_{k}|Y_{k})$ can be rewritten as in Equation (6).

(6) $$p\left(x_{k}|Y_{k}\right)=\frac{p\left(y_{k}|x_{k}\right)p\left(x_{k}|Y_{k-1}\right)}{p(y_{k}|Y_{k-1})}$$

The first term of the nominator represents the likelihood as the measurement PDF of Equation (4), and the second term is the priori PDF, which can be obtained by the Chapman–Kolmogorov equation.

(7) $$\begin{array}{ccc} p(x_{k}|Y_{k-1}) & = & \int p\left(x_{k}|x_{k-1},Y_{k-1}\right)p\left(x_{k-1}|Y_{k-1}\right)dx_{k-1}\\ & = & \int p\left(x_{k}|x_{k-1}\right)p\left(x_{k-1}|Y_{k-1}\right)dx_{k-1} \end{array}$$

The denominator $p(y_{k}|Y_{k-1})$ of Equation (6) is called the evidence and can be calculated by integrating the nominator.

(8) $$p\left(y_{k}|Y_{k-1}\right)=\int p\left(y_{k}|x_{k}\right)p\left(x_{k}|Y_{k-1}\right)dx_{k}$$

That is, $p(x_{k}|Y_{k})$ can be obtained by calculating Equations (6)–(8) for $p(x_{k-1}|Y_{k-1})$.

## 3. PMF with Reliable Time Propagation

### 3.1. Conventional PMF with Direct Time Propagation

To obtain the posteriori PDF in closed-form from Bayesian filtering, the integrals of Equations (7) and (8) must be explicitly calculated. However, it is almost impossible to solve such integrals for general nonlinear models. Therefore, numerical techniques are usually used to obtain approximate solutions, and PMF is one of those approximation methods. The basic concept of PMF is to discretize a state space into equally spaced grids and to calculate a mass (or a weight) in each grid to discretely express the PDF. That is, suppose that the mass $\omega_{k-1|k-1}^{i}$ of the each grid $\xi_{k-1}^{i}$ from the grid set $\Xi_{k-1}\left(N_{k-1}\right)=\left\{\xi_{k-1}^{i};i=1,\cdots ,N_{k-1}\right\}$ defined at time $t_{k-1}$ is as follows.

(9) $$\omega_{k-1|k-1}^{i}\propto p\left(x_{k-1}=\xi_{k-1}^{i}|Y_{k-1}\right)$$

Then, the discretely approximated posteriori PDF at time $t_{k-1}$ can be expressed as Equation (10).

(10) $$\hat{p}\left(x_{k-1}|Y_{k-1}\right)≃\sum_{i=1}^{N_{k-1}}\omega_{k-1|k-1}^{i}\delta \left(x_{k-1}-\xi_{k-1}^{i}\right)$$

where δ(·) is dirac delta function. Previous PMF-related literatures considered the mass $\omega_{k-1|k-1}^{i}$ as the value of the pdf at $\xi_{k-1}^{i}$. Therefore the rectangular volume term $\Delta \xi^{i}$ around $\xi_{k-1}^{i}$ should be included in Equation (10). However, if assuming equal grid intervals and $\sum_{i}\omega_{k-1|k-1}^{i}=1$ for the masses, then the volume is a common term for all masses so the volume can be viewed as included in the mass. This can reduce unnecessary multiplication operations when implementing the algorithm.

When the PDF of Equation (10) is applied to recursive Bayesian filtering, the integral equations of Equations (7) and (8) are converted to discrete summation equations, so that the discretized posteriori PDF at time $t_{k}$ can be obtained in a similar manner. To apply PMF to general nonlinear models, an adaption procedure for irregular grid intervals, grid support, and grid resolution due to nonlinearity of the model must be included. The conventional PMF algorithm including that procedure is as follows [18].
**Algorithm 1** Conventional PMF1: **Initialization** Define the initial grid set and the masses for the initial priori PDF $p(x_{0}|y_{-1})$; $\Xi_{0}\left(N_{0}\right)=\left\{\xi_{0}^{i};i=1,\cdots ,N_{0}\right\}$, $\omega_{0|-1}^{i}=p\left(x_{0}=\xi_{0}^{i}|y_{-1}\right)$. Set k=0.2: **Measurement Update** Calculate the measurement updated masses for all $i=1,\cdots ,N_{k}$ $\omega_{k|k}=c_{k}^{-1}\omega_{k|k-1}p_{v_{k}}\left(y_{k}-h_{k}\left(\xi_{k}^{i}\right)\right)$ $c_{k}=\sum_{i=1}^{N_{k}}\omega_{k|k-1}p_{v_{k}}\left(y_{k}-h_{k}\left(\xi_{k}^{i}\right)\right)$(Normalization Constant)3: **Grid Propagation** Calculate the nonlinear mapped grid set $H_{k+1}\left(N_{k}\right)=\left\{\eta_{k+1}^{i};i=1,\cdots ,N_{k}\right\}$ $\eta_{k+1}^{i}=f_{k}\left(\xi_{k}^{i}\right)$4: **Grid Redefinition** Redefine the grid set $\Xi_{k+1}\left(N_{k+1}\right)=\left\{\xi_{k+1}^{j};j=1,\cdots ,N_{k+1}\right\}$ with regular grid spacing from $H_{k+1}\left(N_{k}\right)$5: **Time Propagation** Calculate the predicted masses for all $j=1,\cdots ,N_{k+1}$ $\omega_{k+1|k}^{j}=\sum_{i=1}^{N_{k}}\omega_{k|k}^{i}p_{w_{k}}\left(\xi_{k+1}^{j}-\eta_{k+1}^{i}\right)$6: Update k:=k+1 and repeat (2)–(5)


Various algorithms can be applied to the grid redefinition of Step (4). Among proposed algorithms, the algorithms suitable for general estimation problems are AGD and BGD proposed by Šimandl [18]. AGD is an algorithm that selects grid support and grid resolution considering the performance of time propagation calculation. AGD assumes Gaussian distribution when selecting grid support, whereas BGD is a grid support selection algorithm that considers non-Gaussian distribution. Suitable algorithms for TRN include the grid resolution/support adaption algorithm considering noise magnitude [19], and the grid support adaption algorithm using mutual information [20]. The density specific grid design algorithm that assumes two different grids [21] and the density difference grid design algorithm based on the differentiation of the PDF in a sparse grid [22] have been presented recently for general estimation problems.

PMF is a global approximation nonlinear filter, so its application range is very wide. However, to apply PMF, we need to know exactly the time evolution model of Equations (1) and (2) or the probabilistic model of Equations (3) and (4). If there are uncertainties in the model or disturbances that cannot be modeled, such terms can be considered as process noise, but the estimation performance may be degraded. The Takagi–Sugeno (T-S) fuzzy affine model is known to be very effective in dealing with such uncertainties and disturbances. As the state-of-the-art result in that field, a sampled-data filtering design technique for I$\hat{t}$o stochastic T-S fuzzy affine system has been proposed recently [31].

### 3.2. PMF with Indirect Time Propagation

If the probability diffusion of the mass through time propagation is not properly processed, only the contraction of the probability distribution by the measurement update is repeated over time. This eventually causes the filter to diverge because the measurement can be no longer reflected in the error correction. Conversely, the excessive diffusion of the probability is the same as losing estimation information so far, which might degrade the estimation performance. That is, in the implementation of filtering, the proper diffusion process is very important for the stability and the performance of the filter.

**Figure 1:**
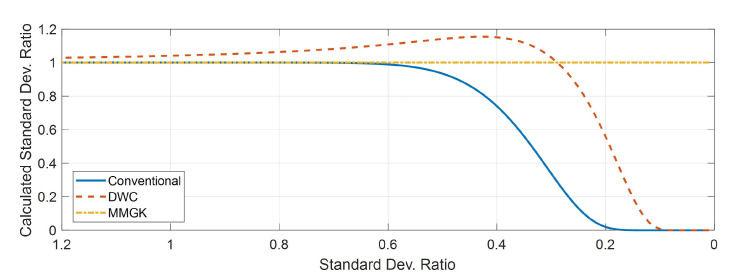
Comparison of the variances of the various kernels, the conventional kernel, MMGK, and DWC.

If the interval between grids is greater than about 1.5 times the standard deviation of the process noise, the kernel for probability diffusion by sampling does not adequately reflect the statistical characteristics of the original noise [23]. VAGK, MMGK, and DWC have been proposed to resolve this problem [23,24,25]. VAGK generation method creates the kernel according to the conventional method and scales up it to match only the variance. So it cannot deal with the process noise whose mean is not zero. MMGK is the structured kernel obtained through linear equations where kernel $K_{t}$ of length *I* matches moments up to I−1. The DWC method uses the difference between the Cumulative Distribution Functions (CDFs) at the upper and lower limits of the volume near the grid. In order to find a kernel generation technique that accurately expresses the variance in the sense of probability diffusion, we compared the variances of the various kernels while varying the variance of the noise. Figure 1 shows the comparison result. The x-axis and y-axis of the graph represent the ratio of the standard deviation of the process noise to the grid interval and the ratio of the variance of the generated kernel to the variance of the noise, respectively. The variance ratio of the conventional kernel gradually decreases as the standard deviation ratio becomes smaller than 0.67. The DWC kernel has a slightly larger value than the original variance when the standard deviation ratio is greater than 0.29 but gradually decreases in the interval less than 0.29. On the other hand, unlike the previous two kernels, MMGK has a variance that exactly matches the original variance regardless of the standard deviation ratio. So we adopt the MMGK as the time propagation kernel.

Adopting MMGK significantly improves the PMF’s inconsistency problem especially when the standard deviation of the process noise is quite small. However, MMGK was proposed to apply to the TRN problem that the system model is an identity matrix. In this paper, to resove the inadequate probability diffusion problem, the general PMF algorithm adopting MMGK is proposed.

**Figure 2:**
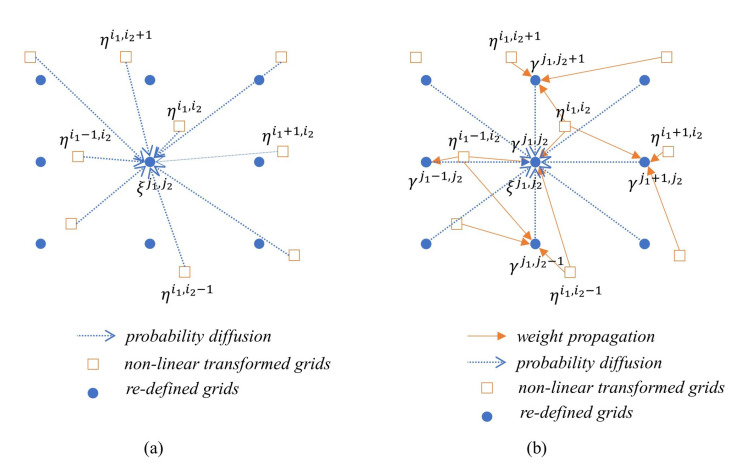
Comparison of the probability diffusion processes between the conventional and the proposed PMF (**a**) Conventional PMF (**b**) Proposed PMF.

MMGK can be applied in the case of regular grid interval, but the kernel in Step (5) of Algorithm 1 is the sampled values of the process noise with the difference between element $\eta_{k+1}^{i}$ of the irregular grid set $H_{k+1}\left(N_{k}\right)$ and element $\xi_{k+1}^{j}$ of the newly defined regular grid set $\Xi_{k+1}\left(N_{k+1}\right)$. Therefore, it is impossible to directly apply MMGK to the conventional PMF algorithm described in the previous section. However, if the convolution operation is performed only on the new grids set $\Xi_{k+1}\left(N_{k+1}\right)$, MMGK can be applied immediately. That is, after redefining mass $\gamma_{k|k}$ for $\xi_{k+1}^{i}$ with $\omega_{k|k}^{i}$, the posteriori mass for grid $\eta_{k+1}^{i}$, the convolution operation is performed indirectly for $\gamma_{k|k}$. $\gamma_{k|k}$ calculation can be easily implemented through various well-known multivariate interpolation algorithms for irregular grids [32]. Figure 2 shows the comparison of the probability diffusion process concepts of the previous method and the proposed one (in order to explain the probability diffusion in the 2D state space, the grid indeces are expressed in 2D).

In the conventional method, the probability values adjusted in proportion to the sampled value of the process noise for all $\eta_{k+1}^{i}$ within the effective distance of process noise near $\xi_{k+1}^{j}$ (for example, 3 times the standard deviation of the process noise) are diffused and accumulated in $\xi_{k+1}^{j}$. On the other hand, the proposed method first calculates the new mass $\gamma_{k|k}^{i}$ for each $\xi_{k+1}^{i}$. A variety of interpolation algorithms can be applied, but in this paper, mass $\gamma_{k|k}^{i}$ is calculated as the linear combination of $\eta_{k+1}^{i}$ around $\xi_{k+1}^{i}$ as follows by applying linear interpolation.

(11) $$\gamma_{k|k}^{i}=\sum_{s\in S_{i}}\lambda_{s,i}\varpi_{k|k}^{s},\;S_{i}=\left\{i^{\prime }|\eta_{k+1}^{i^{\prime }}\;is\;neiborhood\;of\;\xi_{k+1}^{i}\right\},\;\sum_{s\in S_{i}}\lambda_{s,i}=1$$

(12) $$\varpi_{k|k}^{s}=p\left(x_{k+1}=\eta_{k+1}^{s}|Y_{k}\right)=\omega_{k|k}^{s}/{\left|\partial f_{k}\left(x_{k}\right)/\partial x_{k}\right|}_{x_{k}=\xi_{k}^{s}}$$

where $\lambda_{s,i}$ is the linear combination coefficient of $\eta_{k+1}^{s}=f_{k}\left(\xi_{k}^{s}\right)$ concerning for $\xi_{k+1}^{i}$, which satisfies $\xi_{k+1}^{i}=\sum_{s\in S_{i}}\lambda_{s,i}\eta_{k+1}^{s}$ and $\varpi_{k|k}^{s}$ represents the nonlinear transformation of the probability $\omega_{k|k}^{s}$ from $\xi_{k}^{s}$ to $\eta_{k+1}^{s}$. Equation (12) stems from the relationship $f_{Y}\left(y\right)dy=f_{X}\left(x\right)dx$ between two random variables *x* and *y* and the determinant of the Jacobian $\partial f_{k}\left(x_{k}\right)/\partial x_{k}$ for the system model $\eta_{k+1}=f_{k}\left(x_{k}\right)$ represents $d\eta_{k+1}/d\xi_{k}$ [33]. The condition of $\sum_{s\in S_{i}}\lambda_{s,i}=1$ for the coefficient $\lambda_{s,i}$ means that $\xi_{k+1}^{i}$ is a value generated by interpolation. If the interpolation is unavailable, the corresponding mass is assigned as 0. The mass transformation in Equation (12) is only applicable when $f_{k}\left(x_{k}\right)$ is an invertible function such that the solution of $\xi_{k+1}^{i}=f_{k}\left(x_{k}\right)$ is one. If there are multiple solutions, it is necessary to calculate the mass for each solution and add up every transformed mass.

The indirect time propagation operation using the new mass $\gamma_{k|k}^{i}$ by the linear interpolation is shown in Equation (13).

(13) $$\begin{array}{c} \omega_{k+1|k}^{j}=\sum_{i}\gamma_{k|k}^{i}p_{w_{k}}\left(\xi_{k+1}^{j}-\xi_{k+1}^{i}\right) \end{array}$$

The time propagation equation in step (5) of Algorithm 1 and the equation in Equation (13) are mathematically the same. The only difference is that the sampling in Equation (13) is processed on equally spaced grids while the sampling interval of the process noise in step (5) is irregular. Therefore, it is possible to design a structured kernel to accurately reflect the statistics of the process noise. In this paper, MMGK is adopted as the kernel.

The MMGK generation method is as follows [24]. First, let $M_{m,k}$ be the *k*-th moment of the process noise whose mean and variance are $\mu_{m}$ and $\sigma_{m}^{2}$, respectively. For the grids set $\Xi_{k+1}\left(N_{k+1}\right)$ where the grid spacing of the *m*-th state variable is $\Delta \xi_{m}$, considering the effective support of the noise only up to the ±3σ range, it is sufficient to generate a discrete kernel for $x_{s}=s\cdot \Delta \xi_{m}+x_{s,0}$, $s\in [L_{m},\;U_{m}]$, $L_{m}=\left\lfloor \left(\mu_{m}-3\sigma_{m}-x_{s,0}\right)/\Delta \xi_{m}\right\rfloor$, $U_{m}=\left\lceil \left(\mu_{m}+3\sigma_{m}-x_{s,0}\right)/\Delta \xi_{m}\right\rceil$. Let $K_{m}$ be the kernel to be determined, then $\sum_{s=L_{m}}^{U_{m}}x_{s}^{k}K_{m}\left(s\right)=M_{m},k$ has to be satisfied. Therefore, if we write moments from 0-th to $\left(I_{m}-1\right)$-th in vector form, it is as in Equation (14).

(14) $$\begin{array}{c} \left[\begin{array}{ccccc} 1 & 1 & \cdots & 1 & 1\\ x_{L_{m}} & x_{L_{m}+1} & \cdots & x_{U_{m}-1} & x_{U_{m}}\\ x_{L_{m}}^{2} & x_{L_{m}+1}^{2} & \cdots & x_{U_{m}-1}^{2} & x_{U_{m}}^{2}\\ \vdots & \vdots & \ddots & \vdots & \vdots \\ x_{L_{m}}^{I_{m}-1} & x_{L_{m}+1}^{I_{m}-1} & \cdots & x_{U_{m}-1}^{I_{m}-1} & x_{U_{m}}^{I_{m}-1} \end{array}\right]\left[\begin{array}{c} K_{m}\left(L_{m}\right)\\ K_{m}\left(L_{m}+1\right)\\ \vdots \\ K_{m}\left(U_{m}-1\right)\\ K_{m}\left(U_{m}\right) \end{array}\right]\left[\begin{array}{c} 1\\ M_{m,1}\\ \vdots \\ M_{m,I_{m}-2}\\ M_{m,I_{m}-1} \end{array}\right] \end{array}$$

where $I_{m}=U_{m}-L_{m}+1$ is the length of the kernel, and $K_{m}\left(s\right)$ is the element of the kernel. It is the 1-dimension kernel for the process noise. If two dimensional problems are dealt, vector outer product is enought to adopt the MMGK. In this paper, we propose the dimension extended MMGK by outer tensor product. The extended MMGK is a combination of $K_{m}$ generated for each process poise, as shown in Equation (15).

(15) $$\begin{array}{c} Ker=K_{1}\circ K_{2}\circ \cdots \circ \;K_{n} \end{array}$$

where ∘ represents the tensor product(in the sensor of the outer product) [34]. When the generated kernel Ker is applied to the time propagation, the time propagation or the probability diffusion of step (5) for newly defined mass $\gamma_{k|k}^{i}$ as shown in Equation (16).

(16) $$\begin{array}{c} \omega_{k+1|k}^{j}=\sum_{i}\gamma_{k|k}^{i}p_{w_{k}}\left(\xi_{k+1}^{j}-\xi_{k+1}^{i}\right)=\sum_{s\in Ker}\gamma_{k|k}^{j-s}Ker\left(s\right) \end{array}$$

where s=j−i, and s∈Ker means that the index *s* is within the valid range of Ker. Here the mass is represented by the one-dimensional index, but the kernel Ker is the *n*-dimensional tensor. Therefore, to implement the above equation, an appropriate transformation must be included between the 1-dimensional mass index and the *n*-dimensional kernel index. If the mass is expressed and processed with a *n*-dimensional index like the kernel, Equation (16) can be rewritten as Equation (17).

(17) $$\begin{array}{c} \omega_{k+1|k}^{j_{1},j_{2},\cdots ,j_{n}}=\sum_{s_{1}=L_{1}}^{U_{1}}\sum_{s_{2}=L_{2}}^{U_{2}}\cdots \sum_{s_{n}=L_{n}}^{U_{n}}\gamma_{k|k}^{j_{1}-s_{1},j_{2}-s_{2},\cdots ,j_{n}-s_{n}}K_{1}\left(s_{1}\right)K_{2}\left(s_{2}\right)\cdots K_{n}\left(s_{n}\right) \end{array}$$

The proposed time propagation algorithm has the following advantages and disadvantages. In the proposed method, the time propagation performs the convolution through MMGK on grids of equal spacing, so the probability diffusion is more accurate than the conventional one. The conventional method has to calculate the distance between one new grid and all previous grids and examine whether it is within the effective range for the noise probability distribution. On the other hand, the proposed method uses the kernel which considers the effective length of the noise for a new set of grids, so such process is unnecessary. However, the proposed method must additionally perform the interpolation operation for the masses. Bergman’s grid adaption algorithm for TRN is a special case of indirect time propagation that performs only interpolation and decimation of two times intervals [17]. The PMF algorithm including the new indirect time propagation algorithm proposed in this section is as follows.
**Algorithm 2** PMF with Indirect Time Propagation1: **Initialization** Same as (1) of Algorithm 12: **Measurement Update** Same as (2) of Algorithm 13: **Grid Propagation** Same as (3) of Algorithm 14: **Grid and Mass Redefinition** Redefine the grid set $\Xi_{k+1}(N_{k+1}=\left\{\xi_{k+1}^{j};j=1,\cdots ,N_{k+1}\right\}$ from $H_{k+1}\left(N_{k}\right)$ and calculate the interpolated masses for all $\xi_{k+1}^{j}$ $\varpi_{k|k}^{s}=p\left(x_{k+1}=\eta_{k+1}^{s}|Y_{k}\right)=\omega_{k|k}^{s}/{\left|\partial f_{k}\left(x_{k}\right)/\partial x_{k}\right|}_{x_{k}=\xi_{k}^{s}}$ $\gamma_{k|k}^{i}=\sum_{s\in S_{i}}\lambda_{s,i}\varpi_{k|k}^{s}$ If $\eta_{k+1}^{i}=f_{k}\left(x_{k}\right)$ has two or more solutions, repeat the linear interpolation for each solution and calculate their total sum. Calculate the total kernel Ker as a tensor product after finding MMGK $K_{m}$ for each process noise.5: **Time Propagation** Calculate the predicted masses with $\gamma_{k|k}^{i}$ and Ker for all $j=1,\cdots ,N_{k+1}$ $\omega_{k+1|k}^{j}=\sum_{s\in Ker}\gamma_{k|k}^{j-s}Ker\left(s\right)$6: Update k:=k+1 and repeat (2)–(5)


The consistency between the estimation error and the covariance of the filter is very important to ensure the reliable operation of the filter. Specifically, PMF that estimates PDFs by mass on a discrete grid requires consistent handling of the process of probability diffusion through time propagation. However, if the grid interval compared to the variance of the process noise is not dense, the variance of the conventional kernel is treated less than the original design. So, over time, the filter behaves as if it only does measurement updates without time propagation. Eventually, the covariance of the filter gets smaller and smaller, so the measurement update no longer works. However, the proposed PMF adopted MMGK for probability diffusion. MMGK can accurately handle at least the noise variance, so the filter’s consistency can be reliably maintained. Therefore, the performance of the proposed PMF can be improved.

The time propagation of the conventional PMF (step (5) of Algorithm 1) uses the value obtained by directly sampling the process noise for the probability diffusion, and this sampling must be performed $N_{k+1}\times N_{k}$ times. However, the proposed PMF uses MMGK that is independent of the number of grids, and the size of the MMGK is much smaller than the total number of grids, so the computation time of time propagation can be drastically reduced. However, the proposed PMF needs to perform mass interpolation on the new grid set before performing probability diffusion. If the nonlinearity of the system model is too large, the interpolation has to be repeated several times, which can increase the computation time.

### 3.3. Numerical Examples

#### 3.3.1. One Dimensional Growth Model

To verify the performance improvement of the proposed PMF algorithm, a simulation was performed on the following non-stationary growth model used by Gordon et al. in a paper that proposed a bootstrap filter [2,8,26].

(18) $$\begin{array}{c} x_{k+1}=a\cdot x_{k}+b\frac{x_{k}}{1+x_{k}^{2}}+c\cdot \cos\left(k\right)+w_{k}\\ y_{k}=d\cdot x_{k}^{2}+v_{k} \end{array}$$

where each parameter of the model is a=0.5, b=25, c=8, d=0.05, respectively. The measurement noise is $v_{k}∼N\left(0,1^{2}\right)$, and the initial error and the process noise are considered in several cases for performance comparisons. The state variable of the model with the given parameters does not exceed the range [−25, +25] in spite of the process noise so the grid adaption process is not mandatory in this problem. Therefore, the grid set may be fixedly determined according to a predetermined grid interval. In this paper, simulations were performed for a total of four grid intervals. Besides, the same simulation was performed with the bootstrap filter of 1000 particles regardless of the performance change with the grid interval.

**Figure 3:**
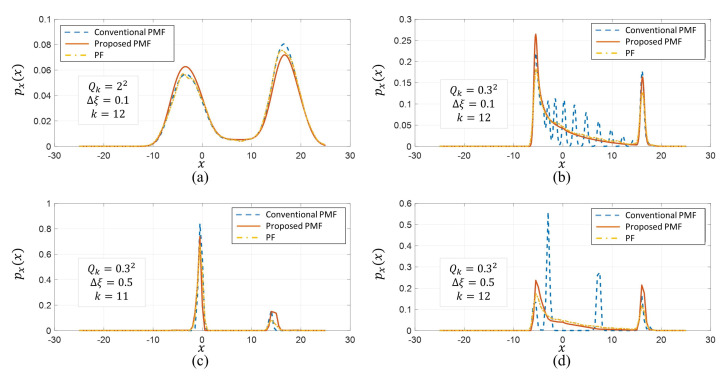
Comparison of the estimated PDFs between the conventional PMF, the proposed PMF, and the PF for several different cases (**a**) $Q_{k}=2^{2},\;\Delta \xi =0.1,\;k=12$ (**b**) $Q_{k}=0.3^{2},\;\Delta \xi =0.1,\;k=12$ (**c**) $Q_{k}=0.3^{2},\;\Delta \xi =0.0.5,\;k=11$ (**d**) $Q_{k}=0.3^{2},\;\Delta \xi =0.0.5,\;k=12$.

First, we compared the PDF outputs estimated by the PMFs and the PF (here, bootstrap filter) for several cases, and Figure 3 shows the results. The pdf of the PMF is just a sampled-function at the grid set. In the case of PF, PDF can be obtained by applying a window function in the form of a normal distribution of small variance to each particle and then summing up all the window functions. The result of k=12 for the case where the initial error and the variance of the process noise are $x_{0}∼N\left(1,5^{2}\right)$ and $Q_{k}=2^{2}$, repectively, and the grid interval Δξ is 0.1 such that the grid interval is sufficiently small compared to the process noise, is shown in (a). As you see, all three algorithms show similar PDFs. In this case, the conventional PMF showed more similar results to the PF. However, in the case of (b) where the process noise variance is reduced to $0.3^{2}$, the conventional PMF shows the completely different aspect of the PDF from the proposed PMF or the PF. In particular, in the range of −5 to +15, the probability distribution of the PF and the proposed PMF shows a moderate decrease trend, whereas the conventional PMF tends to fluctuate. When Δξ is set to 0.5 for $Q_{k}=0.3^{2}$, the PDF estimates for k=11,12 are shown in (c) and (d). In the case of k=11, the three algorithms showed a similar trend, whereas, in the next step, the conventional PMF shows a completely different probability distribution estimate.

**Figure 4:**
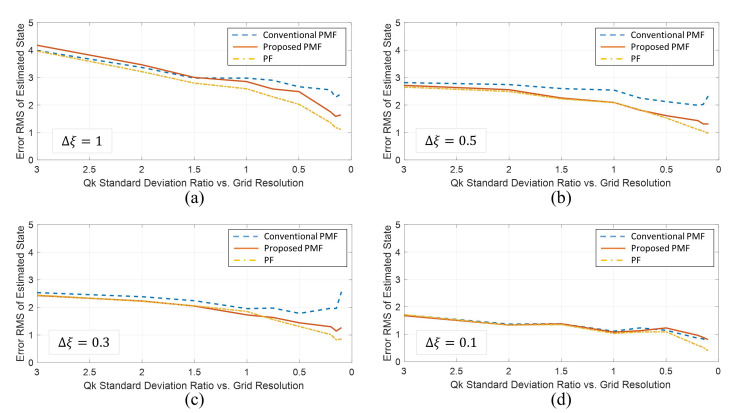
Comparison of the estimation errors between the conventional PMF, the proposed PMF, and the PF for various grid intervals and process noise variances (**a**) Δξ=1 (**b**) Δξ=0.5 (**c**) Δξ=0.3 (**d**) Δξ=0.1.

Next, in the case of $x_{0}∼N\left(5,5^{2}\right)$, the 100 times Monte Carlo simulation results varying the grid interval Δξ and process noise variance $Q_{k}$ are shown in Figure 4. The *x*-axis of each graph is the ratio of process nose standard deviation to grid resolution, which means that the smaller the value, the greater the grid interval than the process noise effective range. The *y*-axis represents the Root Mean Square (RMS) of the estimation error of the simulations. From the results, if the grid interval is sufficiently small (Δξ=0.1), there are no differences in performance regardless of the ratio. On the other hand, In other grid interval cases, if the grid interval is not sufficiently dense($\sqrt{Q_{k}}/\Delta \xi <1$), the performance of the proposed PMF is superior to that of the conventional PMF. In particular, the estimation error of the new PMF tends to decrease like the PF as the ratio decreases, whereas the estimation error of the conventional PMF increases again when $\sqrt{Q_{k}}/\Delta \xi <0.15$. Table 1 shows the numerial results of the simulations. The number of grids of the PMFs for grid intervals 1.0, 0.5, 0.3, and 0.1 were 51, 101, 167, and 501, respectively.

To verify the effect of the computational load reduction of the proposed algorithm, the computation time was measured every epoch. We performed the simulation on the Matlab 2020b single thread environment on Windows 10 operating system with Intel i7-10750H 2.6GHz CPU and 32GB DDR4 Memory. Furthermore, we used vectorized operations and functions to shorten the execution time as much as possible.

**Figure 5:**
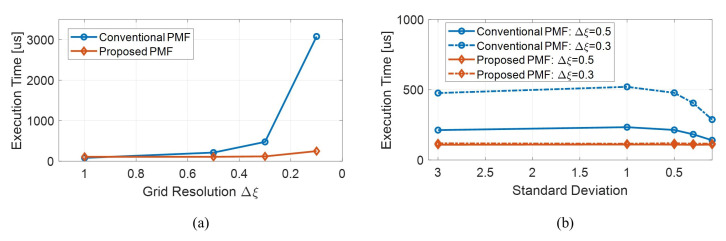
Comparison of the computation time between the conventional PMF and the proposed PMF for various grid intervals and process noise variances (**a**) computation times v.s. grid intervals (**b**) computation time v.s. process noise variances for two grid intervals.

Figure 5 shows the results of measuring the calculation time for various grid intervals and process noises. The result of Figure 5a is the average value of the calculation time for various process noises. In both algorithms, it can be seen that the computational load increases as the grid interval becomes narrower. However, while the time of the proposed algorithm did not change significantly, the time of the conventional one increases exponentially. In particular, when the grid interval is 0.1, the proposed one is about 12.5 times faster than the conventional one. On the other hand, when the grid spacing is 1, the proposed one is about 1.42 times slower. This is because a total of five linear interpolations were performed for mass redefinition considering the high nonlinearity of the growth model. Since mass redefinition process occupies about 70% of the computation time of the proposed algorithm, reducing the number of mass redefinitions can significantly reduce the overall execution time.

Figure 5b shows the calculation time result versus process noise for the case where 0.3 and 0.5 grid intervals. As mentioned earlier, the proposed algorithm did not show a large variation along the grid interval, while the time of the conventional algorithm increased by 2.2 times as the grid interval increased 1.67 times. The time of the proposed algorithm also hardly changes even when the process noise changes, but in the conventional algorithm, the calculation time decreases as the process noise decreases. This is because exponential function calculation is required when performing the probability diffusion due to process noise, and in general numerical calculations, it is treated as 0 if the exponent of the exponential function is less than a certain value.

#### 3.3.2. Two Dimensional Body Fall Problem

As another numerical example for PMF, we performed a simulation for the two dimensional body fall problem. The mathematical model of the body fall problem is as follows [1].

(19) $$\begin{array}{ccc} {\dot{x}}_{1} & = & x_{2}+w_{1}\\ {\dot{x}}_{2} & = & \rho_{0}\exp\left(-x_{1}/k\right)x_{2}^{2}b_{c}/2-g+w_{2}\\ y(t_{k}) & = & \sqrt{M^{2}+{\left(x_{1}\left(t_{k}\right)-a\right)}^{2}}+v_{k} \end{array}$$

As usual, $w_{i}$ is the process noise and and $v_{k}$ is the measurement noise, respectively. The two state variables $x_{1}$ and $x_{2}$ represents the altitude and the velocity of the body, respectively. $\rho_{0}$ is the air density at sea level, *k* is a constant for the relationship between air density and altitude, *g* is the gravity, and $b_{c}$ is the ballistic coefficient. We use a discretized system model with a step size of 100 ms and the measurement is obtained at every 0.5 s. A range measuring device is located at an altitude *a* and the horizontal range between the device and the body is *M*. The constants that we use are given as

$$\begin{array}{ccc} \rho_{0} & = & 105\;\mathrm{kg}\;-\;s^{2}/m^{4}\\ g & = & 9.8\;{m/s}^{2}\\ k & = & 5100\;m\\ b_{c} & = & 6.24\times 10^{-5}\;m^{3}/\mathrm{kg}\;-\;s^{2}\\ M & = & 10,000\;m\\ a & = & 10,000\;m \end{array}$$

The initial conditions of the system and the filter are given as $x_{0}={\left[40,000\;-\;3000\right]}^{T}$, $P_{0}=diag\left(100^{2},\;5^{2}\right)$, $Q_{k}=diag\left(1^{2},\;0.1^{2}\right)$, and $R_{k}=10^{2}$. The grid size of the PMF is 101 × 21 and the simulation time is 30 s. For the first few seconds, the velocity is slowly decreased. However, then the air density increases and drag slows the falling object. Toward the end of the simulation, the body reaches a constant terminal velocity.

**Figure 6:**
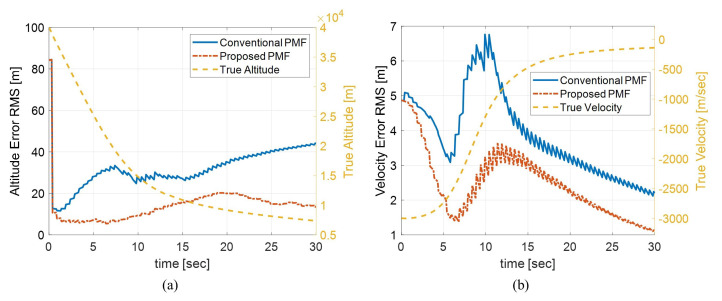
Comparison of the estimation errors between the conventional PMF, the proposed PMF for two dimensional body fall problem (**a**) altitude estimation error (**b**) velocity estimation error.

Figure 6 shows the altitude and velocity RMS errors of 100 times Monte Carlo simulation. The proposed algorithm shows excellent performance in the entire time domain. The altitude error of the proposed algorithm tends to increase slightly between 5 to 20 s interval which the velocity changes rapidly, but after 20 s, the error stably decreases again. On the other hand, the altitude estimation error of the conventional one tends to increase gradually even after 20 s. Likewise, the velocity error increases slightly from 5 s when the velocity starts to change rapidly but stabilizes again after about 12 s. The calculation time was measured under the computing conditions mentioned in 3.3.1. The conventional one took about 30.45 ms for time propagation, but the proposed algorithm took only 5.32 ms. The measurement update of both algorithms took about 0.05 ms. In other words, the proposed algorithm performed about 5.67 times faster than the conventional algorithm while showing superior performance.

It is enough to apply the conventional PMF algorithm if the grid interval is sufficiently small. However, the realtime application of the nonlinear estimation filter might be very limited in computational power depending on the system payload. In that case, the performance is likely to deteriorate when the conventional PMF is applied. On the other hand, in particular, the process noise is small, but if the relative grid interval is not dense sufficiently, it is confirmed through the simulations that the proposed algorithm improves the performance and decreases the computation load over the conventional method. Recently, many studies for the accurate position determination of the small unmanned vehicles such as drones have been conducted, and the estimation models of the localization technique using radio wave, vision, and distance information are generally nonlinear. Therefore, in the sense of the estimation performance and computational load, the proposed algorithm is particularly effective when applied to small vehicles with limited payload in relation to power consumption. Regardless, according to the simulation results, the PF has better performance than the PMFs. However, due to the characteristics of the algorithm, it is known that PMF of the deterministic nature has a superior robustness to PF [35].

## 4. Rao–Blackwellized PMF with Reliable Time Propagation

The algorithmic complexity of PMF is $O(N^{2})$ due to the convolution operation of the time propagation step where *N* is the number of grids. Furthermore, generally, *N* is an exponential function of the dimension *n* of the state variable. So the calculation amount of PMF increases exponentially as *n* increases. The Rao–Blackwellization technique is a representative method to reduce the computational complexity for high-dimensional nonlinear estimation problems based on PF [12,13]. In this technique, when the estimation model can be separated into a nonlinear part and a linear part, nonlinear filtering is applied only to the nonlinear part, and Kalman filtering is applied to the linear part to make the dimension of the nonlinear filter as small as possible. It has been developed and applied to PF earlier, and Šmídl was the first to propose the RBPMF relatively recently [26]. However, since all previous RBPMFs are based on the conventional PMF, there is a problem of degrading the filter stability due to the abnormal probability diffusion for the nonlinear part.

Therefore, in this paper, like the PMF proposed in the previous section, we propose an RBPMF algorithm using MMGK that can more accurately process the probability diffusion. The simulation results to verify the effectiveness of the proposed RBPMF are described. Finally, an RBPMF algorithm with the same indirect time propagation as the PMF proposed in the previous section is proposed for a special case.

### 4.1. Conventional Rao–Blackwellized PMF

Let us consider the following model where the state variable can be separated into a nonlinear part and a linear part [12].

(20) $$\begin{array}{ccc} x_{k}^{n} & = & f_{k-1}^{n}\left(x_{k-1}^{n}\right)+F_{k-1}^{nl}\left(x_{k-1}^{n}\right)x_{k-1}^{l}+w_{k-1}^{n}\\ x_{k}^{l} & = & f_{k-1}^{l}\left(x_{k-1}^{n}\right)+F_{k-1}^{l}\left(x_{k-1}^{n}\right)x_{k-1}^{l}+w_{k-1}^{l}\\ y_{k} & = & h_{k}\left(x_{k}^{n}\right)+H_{k}\left(x_{k}^{n}\right)x_{k}^{l}+v_{k} \end{array}$$

where the state variable, the process noise, and the measurement noise are as follows.

(21) $$\begin{array}{c} x_{k}=\left[\begin{array}{c} x_{k}^{n}\\ x_{k}^{l} \end{array}\right],\;w_{k}=\left[\begin{array}{c} w_{k}^{n}\\ w_{k}^{l} \end{array}\right]∼N\left(0,Q_{k}\right),\;Q_{k}=\left[\begin{array}{cc} Q_{k}^{n} & Q_{k}^{nl}\\ {Q_{k}^{nl}}^{T} & Q_{k}^{l} \end{array}\right],\;v_{k}∼N\left(0,R_{k}\right) \end{array}$$

Then, the posteriori PDF for the decomposed state variable can be divided into two conditional PDFs as shown in Equation (22).

(22) $$\begin{array}{c} p\left(x_{k}|Y_{k}\right)=p\left(x_{k}^{n},x_{k}^{l}|Y_{k}\right)=p\left(x_{k}^{l}|x_{k}^{n},Y_{k}\right)p\left(x_{k}^{n}|Y_{k}\right) \end{array}$$

Here, an important assumption is that the conditional PDF $p(x_{k}^{l}|x_{k}^{n},Y_{k})$ for the linear part $x_{k}^{l}$ given the nonlinear part $x_{k}^{n}$ follows approximately a normal distribution. Therefore, KF is applied to the linear part and PMF is applied only to the PDF estimation of the nonlinear part.

**Figure 7:**
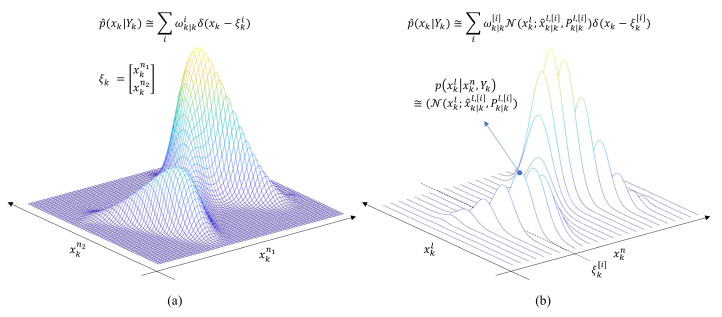
Illustrations of PMF and RBPMF concepts for two dimensional estimation problem (**a**) PMF (**b**) RBPMF.

There are two important aspects from the model in constructing the RBPMF algorithm. First, when processing the nonlinear part, the two terms $F_{k-1}^{nl}\left(x_{k-1}^{n}\right)x_{k-1}^{l}$ and $H_{k}\left(x_{k}^{n}\right)x_{k}^{l}$ due to the linear part are regarded as additional noises. This makes the variances of the effective process and measurement noises be larger when estimating the nonlinear part. Furthermore, in general, the two noises have been considered as zero-mean noise. However, in the case of RBPMF, the effective process and measurement noises are changed to nonzero normal distribution due to the influences of the linear part (see Equations (A4) and (A8)). The second aspect is that the system model for the nonlinear part is processed as an artifact measurement for the linear part and the artifact measurement model must be processed before the system model for the linear part. To understand how these two perspectives are triggered, we described the PDF update procedure for RBPMF divided into six steps in the Appendix A. For the time propagation step for the linear part, if $w_{k}^{n}$ and $w_{k}^{l}$ are correlated with each other, the artifact measurement should be handled carefully, and Schön proposed the well-established algorithm for this [12]. Figure 7 shows an example of a PDF estimated by PMF that treats a state variable as a two-dimensional nonlinear part for a two-dimensional estimation problem, and a PDF estimated by RBPMF composed of a one-dimensional nonlinear part and a one-dimensional linear part.

The conventional RBPMF algorithm based on the Bayesian framework summarized in the Appendix A is as follows.
**Algorithm 3** Conventional RBPMF1: **Initialization** Define initial grids, masses for the nonlinear part PDF $p(x_{0}^{n}|y_{-1})$, and initial normal distributions for linear part PDF $p(x_{0}^{l}|x_{0}^{n},y_{-1})$; $\Xi_{0}\left(N_{0}\right)=\left\{\xi_{0}^{i]};i=1,\cdots ,N_{0}\right\}$, $\omega_{0|-1}^{\left[i\right]}=p\left(x_{0}^{n}=\xi_{0}^{\left[i\right]}|y_{-1}\right)$, $p\left(x_{0}^{l}|x_{0}^{n}=\xi_{0}^{i},y_{-1}\right)=N\left({\hat{x}}_{0|-1}^{l,[i]},P_{0|-1}^{l,[i]}\right)$. Set k=0.2: **Measurement Update for Nonlinear State** Calculate measurement updated masses. $\omega_{k|k}^{\left[i\right]}=c_{k}^{-1}\omega_{k|k-1}^{\left[i\right]}p_{v_{k}^{\prime }}\left(y_{k}-h_{k}\left(\xi_{k}^{\left[i\right]}\right)\right)$ $c_{k}=\sum_{i=1}^{N_{k}}\omega_{k|k-1}^{\left[i\right]}p_{v_{k}^{\prime }}\left(y_{k}-h_{k}\left(\xi_{k}^{\left[i\right]}\right)\right)$ (Normalization Constant) where $v_{i}^{\prime }∼N\left(H_{k}\left(\xi_{k}^{\left[i\right]}\right){\hat{x}}_{k|k-1}^{l,[i]},H_{k}\left(\xi_{k}^{\left[i\right]}\right)P_{k|k-1}^{l,[i]}{H_{k}\left(\xi_{k}^{\left[i\right]}\right)}^{T}+R_{k}\right)$3: **Measurement Update for Linear State** KF measurement update for the linear part with measurement $y_{k}-h_{k}\left(x_{k}^{n}\right)=H_{k}\left(x_{k}^{n}\right)x_{k}^{l}+v_{k}$ $K_{k}^{\left[i\right]}=P_{k|k-1}^{l,[i]}{H_{k}\left(\xi_{k}^{\left[i\right]}\right)}^{T}{\left(H_{k}\left(\xi_{k}^{\left[i\right]}\right)P_{k|k-1}^{l,[i]}{H_{k}\left(\xi_{k}^{\left[i\right]}\right)}^{T}+R_{k}\right)}^{-1}$ $P_{k|k}^{l,[i]}=\left(I-K_{k}^{\left[i\right]}H_{k}\left(\xi_{k}^{\left[i\right]}\right)\right)P_{k|k-1}^{l,[i]}$ ${\hat{x}}_{k|k}^{l,[i]}={\hat{x}}_{k|k-1}^{l,[i]}+K_{k}^{\left[i\right]}\left(y_{k}-h_{k}\left(\xi_{k}^{\left[i\right]}\right)-H_{k}\left(\xi_{k}^{\left[i\right]}\right){\hat{x}}_{k|k-1}^{l,[i]}\right)$4: **Grid Time Propagation for Nonlinear State** Calculate the nonlinear mapped grid set $H_{k+1}\left(N_{k}\right)=\left\{\eta_{k+1}^{\left[i\right]};i=1,\cdots ,N_{k}\right\}$ $\eta_{k+1}^{\left[i\right]}=f_{k}^{n}\left(\xi_{k}^{\left[i\right]}\right)$5: **Grid Redefinition** Redefine the grid set $\Xi_{k+1}\left(N_{k+1}\right)=\left\{\xi_{k+1}^{\left[j\right]};j=1,\cdots ,N_{k+1}\right\}$ from $H_{k+1}\left(N_{k}\right)$6: **Time Propagation for Nonlinear State** Calculate priori masses $\omega_{k+1|k}^{\left[j\right]}$ $\omega_{k+1|k}^{\left[j,i\right]}=p_{w_{i}^{\prime }}\left(\xi_{k+1}^{\left[j\right]}-\eta_{k+1}^{\left[i\right]}\right)\omega_{k|k}^{\left[i\right]}$ $\omega_{k+1|k}^{\left[j\right]}=\sum_{i=1}^{N_{k}}\omega_{k+1|k}^{\left[j,i\right]}$ where $w_{i}^{\prime }∼N\left(F_{k}^{nl}\left(\xi_{k}^{\left[i\right]}\right){\hat{x}}_{k|k}^{l,[i]},F_{k}^{nl}\left(\xi_{k}^{\left[i\right]}\right)P_{k|k}^{l,[i]}{F_{k}^{nl}\left(\xi_{k}^{\left[i\right]}\right)}^{T}+Q_{k}^{n}\right)$7: **Time Propagation for Linear State** KF time propagate for the linear part with artifact measurement $x_{k+1}^{n}-f_{k}^{n}\left(x_{k}^{n}\right)=z_{k}=F_{k}^{nl}\left(x_{k}^{n}\right)x_{k}^{n}+w_{k}^{n}$ ${\hat{x}}_{k+1|k}^{l,[j,i]}=\left({\bar{F}}_{k}^{l,[i]}-L_{k}^{l,[i]}F_{k}^{nl,[i]}\right){\hat{x}}_{k|k}^{l,[i]}+f_{k}^{l}\left(\xi_{k}^{\left[i\right]}\right)+\left({Q_{k}^{nl}}^{T}{Q_{k}^{n}}^{-1}+L_{k}^{l,[i]}\right)z_{k}^{\left[j,i\right]}$ $z_{k}^{\left[j,i\right]}=\xi_{k+1}^{\left[j\right]}-f_{k}^{n}\left(\xi_{k}^{\left[i\right]}\right)$ $P_{k+1|k}^{l,[i]}={\bar{F}}_{k}^{l,[i]}P_{k|k}^{l,[i]}{\bar{F}}_{k}^{l,{\left[i\right]}^{T}}+Q_{k}^{l}-{Q_{k}^{nl}}^{T}{Q_{k}^{n}}^{-1}Q_{k}^{nl}-L_{k}^{l,[i]}N_{k}^{l,[i]}{L_{k}^{l,[i]}}^{T}$ where $F_{k}^{l,[i]}=F_{k}^{l}\left(\xi_{k}^{\left[i\right]}\right)$, $F_{k}^{nl,[i]}=F_{k}^{nl}\left(\xi_{k}^{\left[i\right]}\right)$ and ${\bar{F}}_{k}^{l,[i]}=F_{k}^{l,[i]}-{Q_{k}^{nl}}^{T}{Q_{k}^{n}}^{-1}F_{k}^{nl,[i]}$ $N_{k}^{l,[i]}=F_{k}^{nl,[i]}P_{k|k}^{l,[i]}{F_{k}^{nl,[i]}}^{T}+Q_{k}^{n}$ $L_{k}^{l,[i]}={\bar{F}}_{k}^{l,[i]}P_{k|k}^{l,[i]}F_{k}^{nl,{\left[i\right]}^{T}}{N_{k}^{l,[i]}}^{-1}$8: **Marginalization for Linear State** Marginalize the linear part PDF for $x_{k}^{n}=\xi_{k}^{\left[i\right]}$ by applying moment matching $\alpha_{k+1|k}^{\left[j,i\right]}=\omega_{k+1|k}^{\left[j,i\right]}/\omega_{k+1|k}^{\left[j\right]}$ ${\hat{x}}_{k+1|k}^{l,[j]}=\sum_{i=1}^{N_{k}}\alpha_{k+1|k}^{\left[j,i\right]}{\hat{x}}_{k+1|k}^{l,[j,i]}$ $P_{k+1|k}^{l,[j]}=\sum_{i=1}^{N_{k}}\alpha_{k+1|k}^{\left[j,i\right]}\left(P_{k+1|k}^{l,[i]}+\left({\hat{x}}_{k+1|k}^{l,[j,i]}-{\hat{x}}_{k+1|k}^{l,[j]}\right){\left({\hat{x}}_{k+1|k}^{l,[j,i]}-{\hat{x}}_{k+1|k}^{l,[j]}\right)}^{T}\right)$9: Update k:=k+1 and repeat (2)–(8)


After performing Step (3) or Step (8), the total mean and variance for the linear part can be acquired as shown in Equations (23) and (24).

(23) $${\hat{x}}_{k|k}^{l}=\sum_{i=1}^{N_{k}}\omega_{k|k}^{\left[i\right]}{\hat{x}}_{k|k}^{l,[i]}$$

(24) $$P_{k|k}^{l}=\sum_{i=1}^{N_{k}}\left(P_{k|k}^{l,[i]}+\left({\hat{x}}_{k|k}^{l,[i]}-{\hat{x}}_{k|k}^{l}\right){\left({\hat{x}}_{k|k}^{l,[i]}-{\hat{x}}_{k|k}^{l}\right)}^{T}\right)$$

### 4.2. Rao–Blackwellized PMF with MMGK

In the time propagation of the RBPMF, the probability diffusion is calculated by sampling the normal distributions with mean $F_{k}^{nl}\left(\xi_{k}^{\left[i\right]}\right){\hat{x}}_{k|k}^{l,[i]}$ and variance $F_{k}^{nl}\left(\xi_{k}^{\left[i\right]}\right)P_{k|k}^{l,[i]}{F_{k}^{nl}\left(\xi_{k}^{\left[i\right]}\right)}^{T}+Q_{k}^{n})$ at the grid points. Therefore, if the new grid spacing is not small enough, diffusion might not normally handled as in conventional PMF.

To improve the abnormal probability diffusion process of the RBPMF, the MMGK technique can be applied as in PMF. In PMF, to apply MMGK, the masses of the regular grids were generated through interpolating the masses of the irregular grids. However, in the case of RBPMF, each grid of the nonlinear part is paired with the normal distribution of the individual linear part. That is, even if the nonlinear state space is relocated to the regular grid and the mass interpolation is performed, it is impossible to apply a common kernel because each grid has a individual process noise PDF. Nevertheless, adopting MMGK in RBPMF improves the performance because MMGK can better represent the statistical characteristics of the process noise when the grids are not dense enough.

In addition, the process noise in the time propagation of the nonlinear part is the normal distribution whose mean is no longer zero but $F_{k}^{nl}\left(\xi_{k}^{\left[i\right]}\right){\hat{x}}_{k|k}^{l,[i]}$. However, the mean affects the probability diffusion at the same location as $\eta_{k+1}^{\left[i\right]}=f_{k}^{n}\left(\xi_{k}^{\left[i\right]}\right)$, which corresponds to the nonlinear transformation of the previous grid. Therefore, the process noise can be regarded as the normal distribution with zero mean, and instead, the mean can be reflected in the nonlinear mapping such that it is treated as $\eta_{k+1}^{\left[i\right]}=f_{k}^{n}\left(\xi_{k}^{\left[i\right]}\right)+F_{k}^{nl}\left(\xi_{k}^{\left[i\right]}\right){\hat{x}}_{k|k}^{l,[i]}$. Moreover, it is more advantageous to process the probability diffusion to the redefinition of the the grids in consideration of the movement of the probability distribution by the linear part (note that the process noise has zero mean, but the time propagation is performed by the sampling of the normal distribution of the difference between $\xi_{k+1}^{\left[j\right]}$ and $\eta_{k+1}^{\left[i\right]}$, so the kernel has to be generated for the normal distribution with mean of $\eta_{k+1}^{\left[i\right]}$).

The proposed RBPMF algorithm applying MMGK after grid redefinition considering the linear part effect is as follows.
**Algorithm 4** RBPMF with MMGK1: **Initialization** Same as (1) of Algorithm 32: **Measurement Update for Nonlinear State** Same as (2) of Algorithm 33: **Measurement Update for Linear State** Same as (3) of Algorithm 34: **Grid Propagation for Nonlinear State** Calculate the nonlinear mapped new grid set $H_{k+1}\left(N_{k}\right)=\left\{\eta_{k+1}^{\left[i\right]};i=1,\cdots ,N_{k}\right\}$
$\eta_{k+1}^{\left[i\right]}=f_{k}^{n}\left(\xi_{k}^{\left[i\right]}\right)+F_{k}^{nl}\left(\xi_{k}^{\left[i\right]}\right){\hat{x}}_{k|k}^{l,[i]}$5: **Grid Redefinition** Same as (5) of Algorithm 36: **Time Propagation for Nonlinear State** Generate the MMGK $Ker^{\left[i\right]}$ with mean $\eta_{k+1}^{\left[i\right]}$ and variance $F_{k}^{nl}\left(\xi_{k}^{\left[i\right]}\right)P_{k|k}^{l,[i]}{F_{k}^{nl}\left(\xi_{k}^{\left[i\right]}\right)}^{T}+Q_{k}^{n}$, and calculate the priori masses as follows $\omega_{k+1|k}^{\left[j,i\right]}=\omega_{k|k}^{\left[i\right]}Ker^{\left[i\right]}\left(j\right)$, only for $j\in Ker^{\left[i\right]}$ (otherwise 0) $\omega_{k+1|k}^{\left[j\right]}=\sum_{i=1}^{N_{k}}\omega_{k+1|k}^{\left[j,i\right]}$ where $j\in Ker^{\left[i\right]}$ means that $\xi_{k+1}^{\left[j\right]}$ lies within the effective support range of $Ker^{\left[i\right]}$.7: **Time Propagation for Linear State** Same as (7) of Algorithm 38: **Marginalization for Linear State** Same as (8) of Algorithm 3 except that the summations are conducted only for $\alpha_{k+1|k}^{\left[j,i\right]}\neq 0$9: Update k:=k+1 and repeat (2)–(8)


### 4.3. Rao–Blackwelkized PMF with Indirect Time Propagation for Constant Linear Model Case

Since the RBPMF algorithm for the general nonlinear model has its own covariance matrix for each linear part, it was impossible to apply the indirect time propagation algorithm of Algorithm 2 using one common kernel. However, if $F_{k}^{nl}\left(x_{k}^{n}\right)$, $F_{k}^{l}\left(x_{k}^{n}\right)$, and $H_{k}\left(x_{k}^{n}\right)$ of the model for the linear part are not functions of the non-linear part but constant, then the linear part covariances are approximately equal as follows. Suppose $P_{k|k-1}^{l,[i]}$ are equal for all $i=1,\cdots ,N_{k}$. Then, the calculation results for $P_{k|k}^{l,[i]}$ in Step (3) of Algorithm 4 give the same value by $H_{k}\left(x_{k}^{n}\right)=H_{k}$. Again, the calculation results for $P_{k+1|k}^{l,[i]}$ in Step (7) give the same value by $F_{k}^{nl}\left(x_{k}^{n}\right)=F_{k}^{nl}$, $F_{k}^{l}\left(x_{k}^{n}\right)=F_{k}^{l}$. The last covariance operations of the linear part is the adjustment by the moment matching in Step (8), as shown in Equation (25).

(25) $$\begin{array}{ccc} P_{k+1|k}^{l,[j]} & = & \sum_{i=1}^{N_{k}}\alpha_{k+1|k}^{\left[j,i\right]}\left(P_{k+1|k}^{l,[i]}+\left({\hat{x}}_{k+1|k}^{l,[j,i]}-{\hat{x}}_{k+1|k}^{l,[j]}\right){\left({\hat{x}}_{k+1|k}^{l,[j,i]}-{\hat{x}}_{k+1|k}^{l,[j]}\right)}^{T}\right)\\ & = & P_{k+1|k}^{l}+\sum_{i=1}^{N_{k}}\alpha_{k+1|k}^{\left[j,i\right]}\left({\hat{x}}_{k+1|k}^{l,[j,i]}-{\hat{x}}_{k+1|k}^{l,[j]}\right){\left({\hat{x}}_{k+1|k}^{l,[j,i]}-{\hat{x}}_{k+1|k}^{l,[j]}\right)}^{T} \end{array}$$

Equation (25) has the same covariance $P_{k+1|k}^{l}$ term and the covariance adjustment terms by $N_{k}$ normal distributions scattered from mean ${\hat{x}}_{k+1|k}^{l,[j]}$. Since the covariance adjustment terms act in the direction of making the covariance larger, there is a positive diagonal matrix $\Lambda_{k}^{\left[j\right]}$ that satisfies $P_{k+1|k}^{l,[j]}\leq P_{k+1|k}^{l}+\Lambda_{k}^{\left[j\right]}P_{k+1|k}^{l}\Lambda_{k}^{\left[j\right]}$ (in the sense of positive semi-definite). Then, it can be set as $P_{k+1|k}^{l,[j]}=P_{k+1|k}^{l}+\Lambda_{k}P_{k+1|k}^{l}\Lambda_{k}$, $\Lambda_{k}=\max_{j}\Lambda_{k}^{\left[j\right]}$ by applying a maximum covariance adjustment so that the linear part covariances has the same value for all $j=1,\cdots ,N_{k+1}$ (in the sense of element-wise). Alternatively, $\Lambda_{k}$ can be considered as the tuning parameter of $P_{k+1|k}$. Therefore, by applying the maximum covariance adjustment in moment matching together with the $F_{k}^{nl}\left(x_{k}^{n}\right)=F_{k}^{nl}$, $F_{k}^{l}\left(x_{k}^{n}\right)=F_{k}^{l}$, and $H_{k}\left(x_{k}^{n}\right)=H_{k}$ conditions, the covariance matrices of the linear part are equal. In other words, only one operation for the covariance matrix is enough, which not only can significantly reduce the computational burden but also makes it possible to apply the indirect time propagation algorithm of Algorithm 2 for the nonlinear parts which was not applicable because the covariance matrices are different for each linear part.

The PMF’s indirect time propagation algorithm includes the mass redefinition procedure for the nonlinear state variables. Therefore, to apply it to RBPMF, the linear part redefinition for the new grid must also be performed (the covariance matrix is common, and only the mean corresponding to the state estimate of the linear part is redefined). Linear part redefinition is to redefine $\left(\xi_{k+1}^{\left[i\right]},\gamma_{k|k}^{\left[i\right]},p\left(x_{k}^{l}|x_{k+1}^{n}=\xi_{k+1}^{\left[i\right]},Y_{k}\right)\right)$ pairs from $\left(\xi_{k}^{\left[i\right]},\omega_{k|k}^{\left[i\right]},p\left(x_{k}^{l}|x_{k}^{n}=\xi_{k}^{\left[i\right]},Y_{k}\right)\right)$ pairs according to the nonlinear part redefinition procedure. It has been previously described that $\gamma_{k|k}^{\left[i\right]}$ can be calculated following the same equation as the linear combination of $\xi_{k+1}^{\left[i\right]}$, and the same linear combination can be applied to the linear part redefinition. To show this, examining the PDF of the linear part conditioned on $\xi_{k+1}^{\left[i\right]}$, it is as shown in Equation (26).

(26) $$p\left(x_{k}^{l}|x_{k+1}^{n}=\xi_{k+1}^{\left[i\right]},Y_{k}\right)=\frac{p(x_{k}^{l},x_{k+1}^{n}=\xi_{k+1}^{\left[i\right]},Y_{k})}{p(x_{k+1}^{n}=\xi_{k+1}^{\left[i\right]},Y_{k})}$$

The denominator of Equation (26) is $p\left(x_{k+1}^{n}=\xi_{k+1}^{\left[i\right]},Y_{k}\right)=p\left(x_{k+1}^{n}=\xi_{k+1}^{\left[i\right]}|Y_{k}\right)p\left(Y_{k}\right)=\gamma_{k|k}^{\left[i\right]}p\left(Y_{k}\right)$. The numerator can be calculated through an approximation as follows.

(27) $$\begin{array}{ccc} p(x_{k}^{l},x_{k+1}^{n}=\xi_{k+1}^{\left[i\right]},Y_{k}) & = & p\left(x_{k}^{l},x_{k+1}^{n}=\xi_{k+1}^{\left[i\right]}|Y_{k}\right)p\left(Y_{k}\right)\\ & = & p\left(x_{k}^{l},x_{k+1}^{n}=\sum_{s}\lambda_{s,i}\eta_{k+1}^{\left[s\right]}|Y_{k}\right)p\left(Y_{k}\right) \end{array}$$

**Figure 8:**
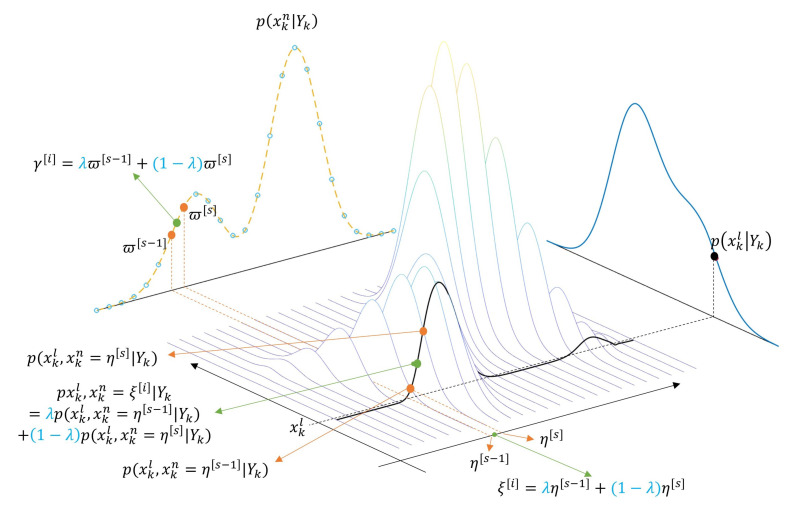
Concept of the approximation of the joint probability of the nonlinear part and the linear part by the linear interpolation in RBPMF.

If we assume a fixed $x_{k}^{l}$, then $x_{k}^{l}$ does not affect an approximation of $x_{k}^{n}$. Therefore, using the linear interpolation coefficients of $\xi_{k+1}^{\left[i\right]}$, Equation (27) can be approximated in the form of the linear interpolation as shown in Equation (28). Figure 8 illustrates the concept of the approximation of the joint probability of the nonlinear part and the linear part by the linear interpolation.

(28) $$\begin{array}{ccc} p(x_{k}^{l},x_{k+1}^{n}=\xi_{k+1}^{\left[i\right]},Y_{k}) & ≃ & \sum_{s}\lambda_{s,i}p\left(x_{k}^{l},x_{k+1}^{n}=\eta_{k+1}^{\left[s\right]}|Y_{k}\right)p\left(Y_{k}\right)\\ & = & \sum_{s}\lambda_{s,i}p\left(x_{k}^{l}|x_{k+1}^{n}=\eta_{k+1}^{\left[s\right]},Y_{k}\right)p\left(x_{k+1}^{n}=\eta_{k+1}^{\left[s\right]}|Y_{k}\right)\\ & = & \sum_{s}\lambda_{s,i}p\left(x_{k}^{l}|x_{k}^{n}=\xi_{k}^{\left[s\right]},Y_{k}\right)\varpi_{k|k}^{\left[s\right]}\\ & = & \sum_{s}\lambda_{s,i}\varpi_{k|k}^{\left[s\right]}N_{x_{k}^{l}}\left({\hat{x}}_{k|k}^{l,[s]},P_{k|k}^{l,[s]}\right) \end{array}$$

Substituting the resulting equation of the numerator and denominator into Equation (26), $p(x_{k}^{l}|x_{k+1}^{n}=\xi_{k+1}^{\left[i\right]},Y_{k})$ becomes Equation (29).

(29) $$p\left(x_{k}^{l}|x_{k+1}^{n}=\xi_{k+1}^{\left[i\right]},Y_{k}\right)=\frac{1}{\sum_{s}\lambda_{s,i}\varpi_{k|k}^{\left[s\right]}}\sum_{s}\lambda_{s,i}\varpi_{k|k}^{\left[s\right]}N_{x_{k}^{l}}\left({\hat{x}}_{k|k}^{l,[s]},P_{k|k}^{l,[s]}\right)$$

Therefore, mean ${\hat{x}}_{k|k}^{l^{\prime },\left[i\right]}$ of $p(x_{k}^{l}|x_{k+1}^{n}=\xi_{k+1}^{\left[i\right]},Y_{k})$ can be obtained as Equation (30).

(30) $$\begin{array}{c} {\hat{x}}_{k|k}^{l^{\prime },\left[i\right]}=\frac{1}{\sum_{s}\lambda_{s,i}\varpi_{k|k}^{\left[s\right]}}\sum_{s}\lambda_{s,i}\varpi_{k|k}^{\left[s\right]}{\hat{x}}_{k|k}^{l,[s]} \end{array}$$

That is, the redefined mean of the linear part can also be calculated as the linear combination of the previous state estimate with the grid redefinition coefficients. If $\varpi_{k|k}^{\left[s\right]}$ has approximately the same value, Equation (30) can be more simplified as Equation (31) because it is $\sum_{s}\lambda_{s,i}=1$.

(31) $$\begin{array}{c} {\hat{x}}_{k|k}^{l^{\prime },\left[i\right]}≃\sum_{s}\lambda_{s,i}{\hat{x}}_{k|k}^{l,[s]} \end{array}$$

If the errors by the redefinition have to be treated, we can model it as an additional process noise and reflect it in the time propagation of the linear part. Although the estimation performance due to the redefinition is slightly deteriorated, the robustness of the linear part filter against model errors can be improved. Figure 8 shows the concept of the state estimate redefinition of the linear part.

The RBPMF algorithm applying the indirect time propagation scheme to the model where $F_{k}^{nl}\left(x_{k}^{n}\right)=F_{k}^{nl}$, $F_{k}^{l}\left(x_{k}^{n}\right)=F_{k}^{l}$, and $H_{k}\left(x_{k}^{n}\right)=H_{k}$ are satisfied is as follows.
**Algorithm 5** RBPMF with Indirect Time Propagation for Constant Linear Model1: **Initialization** Same as (1) of Algorithm 3 except for the constant linear state covariance $P_{0|-1}^{l,[i]}=P_{0|-1}^{l}$2: **Measurement Update for Nonlinear State** Calculate the measurement updated masses. $\omega_{k|k}^{\left[i\right]}=c_{k}^{-1}\omega_{k|k-1}^{\left[i\right]}p_{v_{k}^{\prime }}\left(y_{k}-h_{k}\left(\xi_{k}^{\left[i\right]}\right)\right)$ $c_{k}=\sum_{i=1}^{N_{k}}\omega_{k|k-1}^{\left[i\right]}p_{v_{k}^{\prime }}\left(y_{k}-h_{k}\left(\xi_{k}^{\left[i\right]}\right)\right)$ (Normalization Constant) where $v_{i}^{\prime }∼N\left(H_{k}\left(\xi_{k}^{\left[i\right]}\right){\hat{x}}_{k|k-1}^{l,[i]},H_{k}\left(\xi_{k}^{\left[i\right]}\right)P_{k|k-1}^{l}{H_{k}\left(\xi_{k}^{\left[i\right]}\right)}^{T}+R_{k}\right)$3: **Measurement Update for Linear State** KF measurement updates for the linear part with measurement $y_{k}-h_{k}\left(x_{k}^{n}\right)=H_{k}x_{k}^{l}+v_{k}$ $K_{k}=P_{k|k-1}^{l}{H_{k}}^{T}{\left(H_{k}P_{k|k-1}^{l}{H_{k}}^{T}+R_{k}\right)}^{-1}$ $P_{k|k}^{l}=\left(I-K_{k}^{\left[i\right]}H_{k}\right)P_{k|k-1}^{l}$ ${\hat{x}}_{k|k}^{l,[i]}={\hat{x}}_{k|k-1}^{l,[i]}+K_{k}\left(y_{k}-h_{k}\left(\xi_{k}^{\left[i\right]}\right)-H_{k}{\hat{x}}_{k|k-1}^{l,[i]}\right)$4: **Grid Time Propagation for Nonlinear State** Calculate the nonlinear mapped grid set $H_{k+1}\left(N_{k}\right)=\left\{\eta_{k+1}^{\left[i\right]};i=1,\cdots ,N_{k}\right\}$ $\eta_{k+1}^{\left[i\right]}=f_{k}^{n}\left(\xi_{k}^{\left[i\right]}\right)+F_{k}^{nl}{\hat{x}}_{k|k}^{l,[i]}=f_{k}^{n^{\prime }}\left(\xi_{k}^{\left[i\right]}\right)$5: **Grid, Mass, and Linear State Distribution Redefinition** Redefine the grid set $\Xi_{k+1}\left(N_{k+1}\right)=\left\{\xi_{k+1}^{\left[j\right]};j=1,\cdots ,N_{k+1}\right\}$ from $H_{k+1}\left(N_{k}\right)$. Calculate the interpolated masses $\gamma_{k|k}^{\left[j\right]}$ for $\xi_{k+1}^{\left[j\right]}$ and redefine the linear state mean $\varpi_{k|k}^{\left[s\right]}=p\left(x_{k+1}^{n}=\eta_{k+1}^{\left[s\right]}|Y_{k}\right)=\omega_{k|k}^{\left[s\right]}/{\left|\partial f_{k}^{n^{\prime }}\left(x_{k}^{n}\right)/\partial x_{k}^{n}\right|}_{x_{k}^{n}=\xi_{k}^{\left[s\right]}}$ $\gamma_{k|k}^{\left[i\right]}=\sum_{s}\lambda_{i,s}\varpi_{k|k}^{\left[s\right]}$ ${\hat{x}}_{k|k}^{l^{\prime },\left[i\right]}=\frac{1}{\sum_{s}\lambda_{s,i}\varpi_{k|k}^{\left[s\right]}}\sum_{s}\lambda_{s,i}\varpi_{k|k}^{\left[s\right]}{\hat{x}}_{k|k}^{l,[s]}$6: **Time Propagation for Nonlinear State** Generate the MMGK $K_{j,m}$ for each axis of the process noise whose variance is $F_{k}^{nl}P_{k|k}^{l}{F_{k}^{nl}}^{T}+Q_{k}^{n}$. Calculate the priori masses by applying kernel Ker, the tensor product of $K_{j,m}$ $\omega_{k+1|k}^{\left[j,i\right]}=\gamma_{k|k}^{\left[i\right]}Ker\left(j-i\right)$,  only for *j* where Ker(j−i)≠0 (otherwise 0) $\omega_{k+1|k}^{\left[j\right]}=\sum_{i=1}^{N_{k}}\omega_{k+1|k}^{\left[j,i\right]}$ where the index *i* is limited to the range of $K_{j,m}$7: **Time Propagation for Linear State** KF time propagate for the linear part with artifact measurement $x_{k+1}^{n}-f_{k}^{n}\left(x_{k}^{n}\right)=z_{k}=F_{k}^{nl}x_{k}^{n}+w_{k}^{n}$ ${\hat{x}}_{k+1|k}^{l,[j,i]}=\left({\bar{F}}_{k}^{l}-L_{k}^{l}F_{k}^{nl}\right){\hat{x}}_{k|k}^{l^{\prime },\left[i\right]}+f_{k}^{l}\left(\xi_{k+1}^{\left[i\right]}\right)+\left({Q_{k}^{nl}}^{T}{Q_{k}^{n}}^{-1}+L_{k}^{l}\right)z_{k}^{\left[j,i\right]}$ $z_{k}^{\left[j,i\right]}=\xi_{k+1}^{\left[j\right]}-f_{k}^{n}\left(\xi_{k+1}^{\left[i\right]}\right)$ $P_{k+1|k}^{l}={\bar{F}}_{k}^{l}P_{k|k}^{l}{\bar{F}}_{k}^{l^{T}}+Q_{k}^{l}-{Q_{k}^{nl}}^{T}{Q_{k}^{n}}^{-1}Q_{k}^{nl}-L_{k}^{l}N_{k}^{l}{L_{k}^{l}}^{T}$ where ${\bar{F}}_{k}^{l}=F_{k}^{l}-{Q_{k}^{nl}}^{T}{Q_{k}^{n}}^{-1}F_{k}^{nl}$ $N_{k}^{l}=F_{k}^{nl}P_{k|k}^{l}{F_{k}^{nl}}^{T}+Q_{k}^{n}$ $L_{k}^{l}={\bar{F}}_{k}^{l}P_{k|k}^{l}F_{k}^{nl^{T}}{N_{k}^{l}}^{-1}$8: **Marginalization for Linear State** Marginalize the linear part PDF for $x_{k}^{n}=\xi_{k+1}^{\left[i\right]}$ by applying moment matching $\alpha_{k+1|k}^{\left[j,i\right]}=\omega_{k+1|k}^{\left[j,i\right]}/\omega_{k+1|k}^{\left[j\right]}$ ${\hat{x}}_{k+1|k}^{l,[j]}=\sum_{i=1}^{N_{k}}\alpha_{k+1|k}^{\left[j,i\right]}{\hat{x}}_{k+1|k}^{l,[j,i]}$ $P_{k+1|k}^{l}=P_{k+1|k}^{l}+\Gamma_{k}P_{k+1|k}^{l}\Gamma_{k}$ where $\Gamma_{k}$ is a positive diagonal matrix and the summation is conducted only for $\alpha_{k+1|k}^{\left[j,i\right]}\neq 0$9: Update k:=k+1 and repeat (2)–(8)


As a representative example of such a model, there is Terrain Referenced Navigation (TRN) in which the horizontal position error of two dimensions and the altitude error of one dimension are state variables. For this problem, Peng proposed the interpolation technique of the height errors according to changing grid intervals. However, his method is an index-based adaptive method that simply copies neighbor height error intuitively [30], which is not theoretical and also has a disadvantage that it cannot be applied to changes in grid position. On the other hand, the proposed redefinition algorithm of the linear part is more systematic and can be applied to both the grid position and the interval change.

### 4.4. Numerical Examples

#### 4.4.1. Growth Model with Unknown Parameters

The RBPMF algorithms described in Section 4.1 and Section 4.2 were applied to the simulation model performed in Section 3.3. Although the same model, parameters *b* and *d* are considered as unknown estimation targets. That is, there are three states to be estimated; $x_{k}$, *b*, and *d*. Furthermore, to apply RBPMF, the nonlinear state variable and the linear state variable are set to $x_{k}^{n}=x_{k}$ and $x_{k}^{l}={\left[b,\;d\right]}^{T}$, respectively. Then the model can be rewritten as follows.

(32) $$\begin{array}{ccc} x_{k+1}^{n} & = & a\cdot x_{k}^{n}+\left[\begin{array}{cc} \frac{x_{k}^{n}}{1+{x_{k}^{n}}^{2}} & 0 \end{array}\right]x_{k}^{l}+c\cdot \cos\left(k\right)+w_{k}^{n}\\ x_{k+1}^{l} & = & \;\;\;\;\;\;\;\;\;\;\;\;\;\;\;\;\;\;\;\;\;\;\;\;\;\;\;\;\;\;\;\;\;\;\;\;\;\;\;x_{k}^{l}\;\;\;\;\;\;\;\;\;\;\;\;\;\;\;\;\;\;\;\;\;\;+w_{k}^{l}\\ y_{k} & = & \;\;\;\;\;\;\;\;\;\;\;\;\;\;\;\;\;\;\;\;\;\;\left[\begin{array}{cc} 0 & {x_{k}^{n}}^{2} \end{array}\right]x_{k}^{l}\;\;\;\;\;\;\;\;\;\;\;\;\;\;\;\;\;\;\;\;\;\;+v_{k} \end{array}$$

where the true parameter values are a=0.5, b=5, c=8, and d=0.05 and the initial error, the process noise, and the measurement noise are $x_{0}=∼N\left(5,2^{2}\right)$, $w_{k}^{n}∼N\left(0,0.1^{2}\right)$, $w_{k}^{l}∼N\left(0,diag\left(0.001^{2},0.001^{2}\right)\right)$, and $v_{k}∼N\left(0,0.1^{2}\right)$, respectively. The grid spacing is Δξ=0.5.

**Figure 9:**
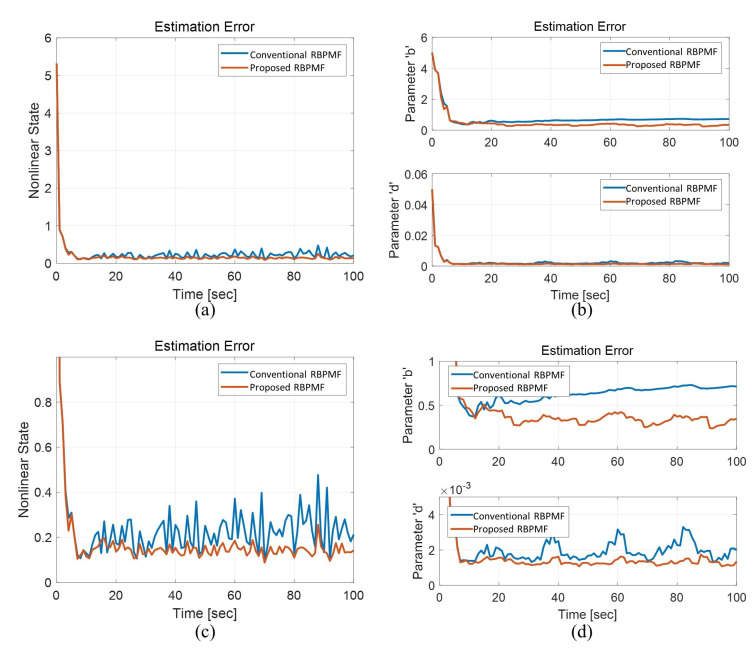
Comparison of the simulation error RMS of the RBPMFs for the growth model with two unknown parameters (**a**) Nonlinear state estimation error (**b**) Parameter estimation errors (**c**) Magnification of (**a**) (**d**) Magnifications of (**b**).

The RMS results of the estimation errors through 100 times Monte Carlo simulations up to 100 s are shown in Figure 9. Sub-figures (c) and (d) are enlarged graphs of the *y*-axis in (a) and (b), respectively. The initial transient characteristics of the conventional RBPMF (Algorithm 3) and the proposed RBPMF (Algorithm 4) are almost same. However, after the convergence, the estimation error of the nonlinear state variable of Algorithm 3 has a large fluctuation, whereas the result of Algorithm 4 shows a much smaller fluctuation. Regarding the parameter estimations, first, the estimation errors of the parameter *b* were approximately 0.7126 and 0.3455 for Algorithm 3 and 4, respectively, and Algorithm 4 showed approximately two times better performance. For the parameter *d*, there is no significant difference in the estimated error magnitude, but the result of Algorithm 3 tends to oscillate at approximately 20 s intervals after 40 s, but no such trend is seen in the result of Algorithm 4.

**Figure 10:**
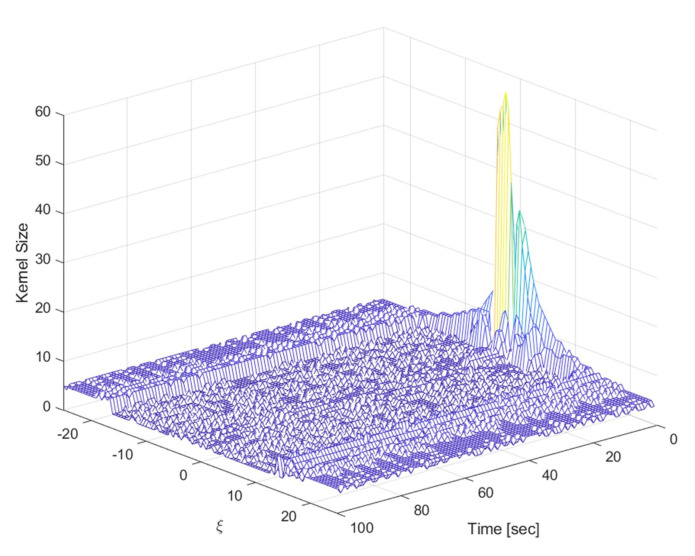
Kernel length profile for the growth model with the two unknown parameters.

Considering the four parameters as the estimation target, since $F_{k}^{nl}\left(x_{k}^{n}\right)$ in Equation (20) is $\left[x_{k}^{n}\;x_{k}^{n}/\left(1+{x_{k}^{n}}^{2}\right)\;\cos\left(k\right)\;0\right]$, the covariance $F_{k}^{nl}\left(\xi_{k}^{\left[i\right]}\right)P_{k|k}^{l,[i]}{F_{k}^{nl}\left(\xi_{k}^{\left[i\right]}\right)}^{T}$ of the process noise of the nonlinear part due to the linear part has a large value compared to the grid interval depending on $x_{k}^{n}$ values. Therefore, in this paper, only the parameter *b* and *d* were estimated, and the simulation was performed only for the problem that the linear part influence on the process noise is small. Figure 10 shows the change in kernel length for MMGK creation over time for one of the previous simulations. Except for the initial transient region, the kernel length within the range of ξ<|15| maintains 3 to 4 for almost all regions, and in this case, the performance is improved by applying MMGK.

#### 4.4.2. Tightly-Coupled INS/TRN Integration

TRN is the most representative application field of PMF. The basic concept of TRN is to find the position by comparing the difference between the absolute altitude of the Inertial Navigation System (INS) output and the relative altitude of the Radio Altimeter (RA) with a terrain elevation database. TERCOM, the first TRN system, is based on a batch processing algorithm that intermittently estimates the position by accumulating measured values for a certain period of time, but in recent years it is gradually developing into a filter-based sequential processing method. Since the measurement model of TRN is the terrain itself and the terrain has very high nonlinearity, it is mandatory to apply a nonlinear filter to the sequential processing TRN algorithm. In general, TRN is integrated with INS and there are three ways to INS/TRN integration as follows.

- No Integration: Single TRN filter structure without any integration;
- Loosely-coupled: Cascaded structure of the INS aiding filter following TRN filter;
- Tightly-coupled: Single filter structure combining TRN filter and INS aiding filter.

In this paper, the proposed RBPMF algorithm is applied to the tightly-coupled method, which is known to have the best performance among the three methods. To apply RBPMF, first, the mathematical model of TRN must be constructed as shown in Equation (20). Here, a 15th order model including all of the position errors, altitude errors, velocity errors, attitude errors, accelerometer bias errors, and gyro bias errors is considered. Among the 15th state variables, only the two horizontal position errors, which are the independent variable of the terrain elevation function, is selected as the nonlinear part state variable, and the rest is the linear part state variable. Based on this, the tightly-coupled INS/TRN system model is represented as follows.

(33) $$\begin{array}{ccc} x_{k}^{n} & = & I_{2\times 2}x_{k-1}^{n}\;\;+I_{2\times 13}x_{k-1}^{l}+w_{k-1}^{n}\\ x_{k}^{l} & = & \;\;\;\;\;\;\;\;\;\;\;\;\;\;\;\;\;\;\;\;\;\;F_{k-1}^{l}x_{k-1}^{n}\;\;+w_{k-1}^{l} \end{array}$$

(34) $$\begin{array}{ccc} y_{k} & = & h_{DEM}\left(x_{k}^{n}\right)-I_{1\times 13}x_{k}^{l}\;\;\;\;+v_{k} \end{array}$$

A detailed explanation of the INS error model $F_{k-1}^{l}$ is provided in many other pieces of literature, so it is omitted in this paper. Generally, the horizontal position errors are generally expressed as the angular errors of the latitude and the longitude, but in that case, the system model for the nonlinear part is not a unit matrix because it is affected by the earth radii. Therefore, in this paper, the horizontal position errors are considered as a distance error instead of the angle.

**Figure 11:**
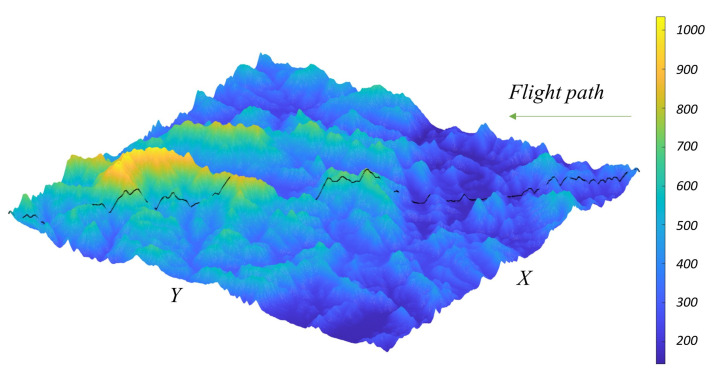
Ground trajectory and terrain elevation for INS/TRN simulation.

The configuration of various conditions for simulation is as follows. First, the PMF grid size is 51 × 51. Time propagation and measurement update are performed every 1 s. Due to the characteristics of RBPMF, the nonlinear part is changed by the velocity error of the linear part, so the grid redefinition is performed every time propagation. The initial grid interval is set to cover the 3-sigma region of the initial position error of the INS, and the mass is initialized in the form of a normal distribution. Various initial errors and sensor errors are summarized in Table 2. Terrain elevation data with about 30m resolutions is used for simulation. We conducted 50 times Monte Carlo simulation to obtain position error RMS every time. The flight trajectory is assumed to be straight at constant speed for 200 s. Figure 11 shows the ground trajectory on the terrain elevation. The flight altitude is 300m higher than the highest ground altitude below the flight trajectory, and it is assumed that there is no INS altitude errors and vertical velocity error.

**Figure 12:**
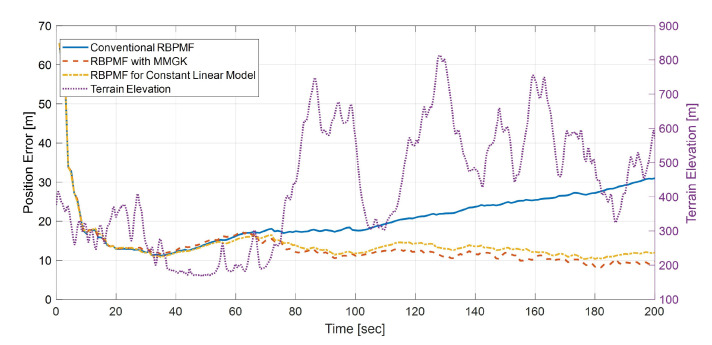
Comparison of the position error RMS of the conventional RBPMF (Algorithm 3), RBPMF with MMGK (Algorithm 4), and RBPMF for constant linear model (Algorithm 5).

Figure 12 is a graph of the position error of INS/TRN based on three RBPMFs. Up to the first 60 s, the three algorithms show almost the same position error results. However, after 60 s, only the two proposed algorithms show a similar positional error, and after about 80 s, the stable positional errors of 10 m level are shown in a rough terrain region which is suitable for TRN operation. On the other hand, the error of the conventional RBPMF increased up to the 30 m level as the error was not bound and gradually diverged after 80 s.

Table 3 summarizes the execution times of the three algorithms. It can be seen that the proposed algorithms operate several times faster than the conventional algorithm. In particular, Algorithm 5 is found to be about 5.67 times faster. Algorithm 4 computes the linear covariance as many as the number of grids, whereas Algorithm 5 computes only one covariance. Due to this effect, the execution time of Algorithm 5 decreased by 29.3 ms and 16.5 ms, respectively, in time propagation and measurement update time compared to Algorithm 4. In other words, Algorithm 5 is calculated approximately 1.35 times faster than Algorithm 4.

## 5. Conclusions

In this paper, we proposed the various algorithms that can improve the reliability of the time propagation of PMF. First, we proposed the PMF algorithm that indirectly performs the probability diffusion through the mass redefinition and the dimension extended MMGK, as opposed to the conventional PMF directly sampling the process noise to perform the probability diffusion. The proposed PMF outperforms the conventional one but requires less computation load. To verify the performance of the proposed algorithm, the simulation was performed on the Growth model and body fall problem. The simulation results show that the proposed PMF performance is improved under most conditions and the computational load is reduced by up to 12 times. RBPMF is one remedy to resolve the excessive computational burden of PMF, which increases exponentially as the dimension of the state variable increases. However, the RBPMF based on the conventional PMF has the same problem in the probability diffusion process. So, as the second result of this paper, we proposed the RBPMF algorithm adopting MMGK but without mass redefinition. The third result is the proposal of the RBPMF algorithm including the redefinition step of the linear part for indirect time propagation in the case of a constant linear model such as TRN. Simulations results for the Growth model with two unknown parameters and the tighlty-coupled INS/TRN integration of the 15th order state variable verify that the proposed algorithm shows better performance with less computation than the conventional RBPMF.

When generating the extended MMGK, we ignored correlations between process noises. However, to consider the correlations, higher moments for multivariate normal distribution have to be dealt with. The proposed algorithms perform mass linear interpolation and it requires the nonlinear transformation of the PDF. Its implementation might not be easy if the system dynamics model is complicated and it has multi-solutions for a given target value.

## Figures and Tables

**Table 1:** Estimation errors of the conventional PMF, the proposed PMF, and PF for various grid intervals and process noise variances.

Grid Resolutions	Algorithms	Process Noise Standard Deviation Ratio vs. Grid Resolution								
		3.0	2.0	1.5	1.0	0.75	0.5	0.2	0.15	0.1
1.0	Conventional PMF	3.998	3.113	2.924	3.018	2.789	2.773	2.512	2.399	2.334
	Proposed PMF	4.153	3.391	3.052	2.918	2.458	2.469	1.689	1.684	1.618
	Bootstrap PF	4.059	3.077	2.760	2.644	2.259	2.125	1.379	1.233	1.187
0.5	Conventional PMF	2.815	2.744	2.598	2.543	2.250	2.122	1.990	2.022	2.345
	Proposed PMF	2.715	2.557	2.254	2.091	1.810	1.611	1.431	1.311	1.311
	Bootstrap PF	2.652	2.493	2.222	2.089	1.821	1.525	1.107	1.049	0.968
0.3	Conventional PMF	2.535	2.387	2.242	1.955	1.975	1.783	1.969	1.959	2.567
	Proposed PMF	2.431	2.231	2.048	1.728	1.634	1.441	1.299	1.144	1.265
	Bootstrap PF	2.446	2.221	2.058	1.852	1.557	1.307	1.013	0.826	0.852
0.1	Conventional PMF	1.709	1.369	1.381	1.111	1.230	1.144	0.858	0.835	0.744
	Proposed PMF	1.678	1.336	1.379	1.078	1.128	1.229	0.963	0.890	0.805
	Bootstrap PF	1.713	1.334	1.352	1.029	1.083	1.087	0.598	0.530	0.399

**Table 2:** Initial navigation and sensor error configuration for tightly-coupled INS/TRN simulation.

Error Types		Error Magnitude (Standard Deviation)
Initial Navigation Errors	Positions	50/50/5 m (δλ/δφ/δh)
	Velocities	0.3/0.3/0.1 m/sec ($\delta v_{e}$/$\delta v_{n}$/$\delta v_{u}$)
	Attitudes	0.1/0.1/1 mrad ($\phi_{e}$/$\phi_{n}$/$\phi_{u}$)
Accelrometer Bias Error		100 ug
Accelrometer White Noise		10 ug
Gyro Bias Error		0.005 deg/hr
Gyro White Noise		0.005 $deg/\sqrt{hr}$
Radar Altimeter Error		10 m

**Table 3:** Comparison of the average computation time per epoch of the RBPMF algorithms (unit: ms).

Algorithm	Propagation	Update	Total	Ratio
Conventional RBPMF (Algorithm 3)	723	28.5	751.5	1
RBPMF with MMGK (Algorithm 4)	152.5	25.8	178.3	4.21
RBPMF for Constant Linear Model (Algorithm 5)	123.2	9.3	132.5	5.67

## Appendix A

First, for the random vector whose PDF is $p_{w}\left(w\right)=N_{w}\left(m_{w},P_{w}\right)$, the following equation holds.

(A1) $$p_{w}\left(f\left(x\right)-Ay\right)N_{y}\left(m_{y},P_{y}\right)=p_{z}\left(f\left(x\right)\right)N_{y}\left(m,P\right)$$

where *m* and *P* are $m=P(A^{T}P_{w}^{-1}f\left(x\right)+P_{y}^{-1}m_{y})$, $P^{-1}=A^{T}P_{w}^{-1}A+P_{y}^{-1}$, respectively, and the new random variable *z* follows the normal distribution whose mean and covariance are $m_{w}+Am_{y}$ and $P_{w}+AP_{y}A^{T}$, respectively [36].

The Rao–Blackwellized Bayesian filtering algorithm consists of 6 steps to obtain $p(x_{k+1}^{l}|x_{k+1}^{n},Y_{k})$ from $p(x_{k}^{l}|x_{k}^{n},Y_{k-1})$.

(1) $p\left(x_{k}^{n}|Y_{k-1}\right)\to p\left(x_{k}^{n}|Y_{k}\right)$

Applying the Bayes’ rule to $p(x_{k}^{n}|Y_{k})$ is as follows.

(A2) $$p\left(x_{k}^{n}|Y_{k}\right)=p\left(x_{k}^{n}|y_{k},Y_{k-1}\right)=\frac{y_{k}\left|x_{k}^{n},Y_{k-1}\right)p\left(x_{k}^{n}|Y_{k-1}\right)}{p(y_{k}|Y_{k-1})}$$

The first term of the numerator is likelihood, which can be obtained as follows.

(A3) $$\begin{array}{ccc} p(y_{k}|x_{k}^{n},Y_{k-1}) & = & \;\int \int \;p\left(y_{k}|x_{k}^{l},x_{k}^{n},v_{k},Y_{k-1}\right)p\left(x_{k}^{l}|x_{k}^{n},Y_{k-1}\right)p\left(v_{k}\right)dv_{k}dx_{k}^{l}\\ & = & \;\int \int \;\delta \left(y_{k}-h_{k}\left(x_{k}^{n}\right)-H_{k}\left(x_{k}^{n}\right)x_{k}^{l}-v_{k}\right)p\left(x_{k}^{l}|x_{k}^{n},Y_{k-1}\right)p\left(v_{k}\right)dv_{k}dx_{k}^{l}\\ & = & \int p_{v_{k}}\left(y_{k}-h_{k}\left(x_{k}^{n}\right)-H_{k}\left(x_{k}^{n}\right)x_{k}^{l}\right)p\left(x_{k}^{l}|x_{k}^{n},Y_{k-1}\right)dx_{k}^{l} \end{array}$$

Since $p_{v_{k}}\left(v_{k}\right)=N_{v_{k}}\left(m_{v,k},R_{k}\right)$ and $p\left(x_{k}^{l}|x_{k}^{n},Y_{k-1}\right)=N_{x_{k}^{l}}\left({\hat{x}}_{k|k-1}^{l}\left(x_{k}^{n}\right),P_{k|k-1}^{l}\left(x_{k}^{n}\right)\right)$ are also normal distribution, it can be rewritten as follows by Equation (A1).

(A4) $$\begin{array}{ccc} p(y_{k}|x_{k}^{n},Y_{k-1}) & = & \int p_{v^{\prime }}\left(y_{k}-h_{k}\left(x_{k}^{n}\right)\right)N_{x_{k}^{l}}\left(m,P\right)dx_{k}^{l}\\ & = & p_{v^{\prime }}\left(y_{k}-h_{k}\left(x_{k}^{n}\right)\right) \end{array}$$

where $v^{\prime }∼N\left(m_{v,k}+H_{k}\left(x_{k}^{n}\right){\hat{x}}_{k|k-1}^{l},H_{k}\left(x_{k}^{n}\right)P_{k|k-1}^{l}{H_{k}\left(x_{k}^{n}\right)}^{T}+R_{k}\right)$ and *m*, *P* are $m=P({H_{k}\left(x_{k}^{n}\right)}^{T}$${R_{k}}^{-1}\left(y_{k}^{n}-h_{k}\left(x_{k}^{n}\right)\right)+P_{k|k-1}^{l^{-1}}{\hat{x}}_{k|k-1}^{l})$, $P^{-1}={H_{k}\left(x_{k}^{n}\right)}^{T}{R_{k}}^{-1}H_{k}\left(x_{k}^{n}\right)+P_{k|k-1}^{l^{-1}}$, respectively.

(2) $p\left(x_{k}^{l}|x_{k}^{n},Y_{k-1}\right)\to p\left(x_{k}^{l}|x_{k}^{n},Y_{k}\right)$

Applying the Bayes’ rule to $p(x_{k}^{l}|x_{k}^{n},Y_{k})$ is as follows.

(A5) $$p(x_{k}^{l}|x_{k}^{n},y_{k},Y_{k-1})=\frac{p\left(y_{k}|x_{k}^{l},x_{k}^{n},Y_{k-1}\right)p\left(x_{k}^{l}|x_{k}^{n},Y_{k-1}\right)}{p(y_{k}|x_{k}^{n},Y_{k-1})}$$

The first term in the numerator is likelihood, which is as follows.

(A6) $$p\left(y_{k}|x_{k}^{l},x_{k}^{n},Y_{k-1}\right)=p_{v_{k}}\left(y_{k}-h_{k}\left(x_{k}^{n}\right)-H_{k}\left(x_{k}^{n}\right)x_{k}^{l}\right)$$

That is, since it is the likelihood for the linear measurement model $H_{k}\left(x_{k}^{n}\right)$ and the measurement $y_{k}-h_{k}\left(x_{k}^{n}\right)$ and $p(x_{k}^{l}|x_{k}^{n},Y_{k-1})$ is the normal distribution, the measurement update for the linear part, which gives $p\left(x_{k}^{l}|x_{k}^{n},Y_{k}\right)=N_{x_{k}^{l}}\left({\hat{x}}_{k|k}^{l}\left(x_{k}^{n}\right),P_{k|k}^{l}\left(x_{k}^{n}\right)\right)$, can be performed by applying KF.

(3) $p\left(x_{k}^{n}|Y_{k}\right)\to p\left(x_{k+1}^{n}|Y_{k}\right)$

$p(x_{k+1}^{n}|Y_{k})$ is as follows from the Chapman–Kolmogorov equation.

(A7) $$\begin{array}{ccc} p(x_{k+1}^{n}|Y_{k}) & = & \;\int \int \int \;p\left(x_{k+1}^{n}|x_{k}^{n},x_{k}^{l},w_{k}^{n},Y_{k}\right)p\left(x_{k}^{n},x_{k}^{l},w_{k}^{n}|Y_{k}\right)dw_{k}^{n}dx_{k}^{l}dx_{k}^{n}\\ & = & \;\int \int \int \;\delta \left(x_{k+1}^{n}-f_{k}^{n}\left(x_{k}^{n}\right)-F_{k}^{n}\left(x_{k}^{n}\right)x_{k}^{l}-w_{k}^{n}\right)p\left(x_{k}^{n},x_{k}^{l}|Y_{k}\right)p\left(w_{k}^{n}\right)dw_{k}^{n}dx_{k}^{l}dx_{k}^{n}\\ & = & \;\int \int \int \;\delta \left(x_{k+1}^{n}-f_{k}^{n}\left(x_{k}^{n}\right)-F_{k}^{n}\left(x_{k}^{n}\right)x_{k}^{l}-w_{k}^{n}\right)p\left(w_{k}^{n}\right)dw_{k}^{n}p\left(x_{k}^{l}|x_{k}^{n},Y_{k}\right)p\left(x_{k}^{n}|Y_{k}\right)dx_{k}^{l}dx_{k}^{n}\\ & = & \;\int \int \;p_{w_{k}^{n}}\left(x_{k+1}^{n}-f_{k}^{n}\left(x_{k}^{n}\right)-F_{k}^{n}\left(x_{k}^{n}\right)x_{k}^{l}\right)p\left(x_{k}^{l}|x_{k}^{n},Y_{k}\right)p\left(x_{k}^{n}|Y_{k}\right)dx_{k}^{l}dx_{k}^{n} \end{array}$$

Since $p_{w_{k}^{n}}\left(w_{k}^{n}\right)=N_{w_{k}^{n}}\left(m_{w,k},Q_{k}^{n}\right)$ and $p\left(x_{k}^{l}|x_{k}^{n},Y_{k}\right)=N_{x_{k}^{l}}\left({\hat{x}}_{k|k}^{l}\left(x_{k}^{n}\right),P_{k|k}^{l}\left(x_{k}^{n}\right)\right)$ are also normal distribution, it can be rewritten as follows by Equation (A1).

(A8) $$\begin{array}{ccc} p(x_{k+1}^{n}|Y_{k}) & = & \;\int \int \;p_{w^{\prime }}\left(x_{k+1}^{n}-f_{k}^{n}\left(x_{k}^{n}\right)\right)N_{x_{k}^{l}}\left(m,P\right)p\left(x_{k}^{n}|Y_{k}\right)dx_{k}^{l}dx_{k}^{n}\\ & = & \int p_{w^{\prime }}\left(x_{k+1}^{n}-f_{k}^{n}\left(x_{k}^{n}\right)\right)p\left(x_{k}^{n}|Y_{k}\right)\int N_{x_{k}^{l}}\left(m,P\right)dx_{k}^{l}dx_{k}^{n}\\ & = & \int p_{w^{\prime }}\left(x_{k+1}^{n}-f_{k}^{n}\left(x_{k}^{n}\right)\right)p\left(x_{k}^{n}|Y_{k}\right)dx_{k}^{n} \end{array}$$

where $w^{\prime }∼N(m_{w,k}^{n}+F_{k}^{n}\left(x_{k}^{n}\right){\hat{x}}_{k|k}^{l},Q_{w,k}^{n}+F_{k}^{n}\left(x_{k}^{n}\right)P_{k|k}^{l}F_{k}^{n}{\left(x_{k}^{n}\right)}^{T}$ and *m*, *P* are $m=P(F_{k}^{n}{\left(x_{k}^{n}\right)}^{T}$$Q_{k}^{n^{-1}}\left(x_{k+1}^{n}-f_{k}^{n}\left(x_{k}^{n}\right)\right)+P_{k|k}^{l^{-1}}{\hat{x}}_{k|k}^{l})$, $P^{-1}=F_{k}^{n}{\left(x_{k}^{n}\right)}^{T}Q_{k}^{n^{-1}}F_{k}^{n}\left(x_{k}^{n}\right)+P_{k|k}^{l^{-1}}$, respectively.

(4) $p\left(x_{k}^{l}|x_{K}^{n},Y_{k}\right)\to p\left(x_{k}^{l}|x_{k}^{n},x_{k+1}^{n},Y_{k}\right)$

Applying Bayes’s rule to $p(x_{k}^{l}|x_{k}^{n},x_{k+1}^{n},Y_{k})$ is as follows.

(A9) $$\begin{array}{ccc} p(x_{k}^{l}|x_{k}^{n},x_{k+1}^{n},Y_{k}) & = & \frac{p\left(x_{k+1}^{n}|x_{k}^{l},x_{k}^{n},Y_{k}\right)p\left(x_{k}^{l}|x_{k}^{n},Y_{k}\right)p\left(x_{k}^{n}|Y_{k}\right)}{p\left(x_{k+1}^{n}|x_{k}^{n},Y_{k}\right)p\left(x_{k}^{n}|Y_{k}\right)}\\ & = & \frac{p\left(x_{k+1}^{n}|x_{k}^{l},x_{k}^{n},Y_{k}\right)p\left(x_{k}^{l}|x_{k}^{n},Y_{k}\right)}{p(x_{k+1}^{n}|x_{k}^{n},Y_{k})} \end{array}$$

The first term in the numerator is likelihood, which is as follows.

(A10) $$\begin{array}{ccc} p(x_{k+1}^{n}|x_{k}^{l},x_{k}^{n},Y_{k}) & = & \int p\left(x_{k+1}^{n}|x_{k}^{l},x_{k}^{n},w_{k}^{,}Y_{k}\right)p\left(w_{k}^{n}\right)dw_{k}^{n}\\ & = & p_{w_{k}^{n}}\left(x_{k+1}^{n}-f_{k}^{n}\left(x_{k}^{n}\right)-F_{k}^{n}\left(x_{k}^{n}\right)x_{k}^{l}\right) \end{array}$$

That is, since the above equation is the likelihood for linear measurement model $F_{k}^{n}\left(x_{k}^{n}\right)$ and the measurement $x_{k+1}^{n}-f_{k}^{n}\left(x_{k}^{n}\right)$ and $p(x_{k}^{l}|x_{k}^{n},Y_{k})$ is normal distribution, KF can be applied to perform the artifact measurement update for the linear part.

(5) $p\left(x_{k}^{l}|x_{k}^{n},x_{k+1}^{n},Y_{k}\right)\to p\left(x_{k+1}^{l}|x_{k}^{n},x_{k+1}^{n},Y_{k}\right)$

$p(x_{k+1}^{l}|x_{k}^{n},x_{k+1}^{n},Y_{k})$ is as follows from the Chapman–Kolmogorov equation.

(A11) $$\begin{array}{ccc} p(x_{k+1}^{l}|x_{k}^{n},x_{k+1}^{n},Y_{k}) & = & \;\int \int \;p\left(x_{k+1}^{l}|x_{k}^{l},x_{k}^{n},x_{k+1}^{n},w_{k}^{l},Y_{k}\right)p\left(x_{k}^{l},w_{k}^{l}|x_{k}^{n},x_{k+1}^{n},Y_{k}\right)dw_{k}^{l}dx_{k}^{l}\\ & = & \;\int \int \;p\left(x_{k+1}^{l}|x_{k}^{l},x_{k}^{n},x_{k+1}^{n},w_{k}^{l}\right)p\left(x_{k}^{l}|x_{k}^{n},x_{k+1}^{n},Y_{k}\right)p\left(w_{k}^{l}\right)dw_{k}^{l}dx_{k}^{l}\\ & = & \int p\left(x_{k}^{l}|x_{k}^{n},x_{k+1}^{n},Y_{k}\right)\int \delta \left(x_{k+1}^{l}-f_{k}^{l}\left(x_{k}^{n}\right)-F_{k}^{l}\left(x_{k}^{n}\right)x_{k}^{l}-w_{k}^{l}\right)p\left(w_{k}^{l}\right)dw_{k}^{l}dx_{k}^{l}\\ & = & \int p\left(x_{k}^{l}|x_{k}^{n},x_{k+1}^{n},Y_{k}\right)p_{w_{k}^{l}}\left(x_{k+1}^{l}-f_{k}^{l}\left(x_{k}^{n}\right)-F_{k}^{l}\left(x_{k}^{n}\right)x_{k}^{l}\right)dx_{k}^{l} \end{array}$$

Considering $f_{k}^{l}\left(x_{k}^{n}\right)$ as the known input for model $x_{k}^{l}$, since the update equation from $x_{k}^{l}$ to $x_{k+1}^{l}$ is the linear model and $p(x_{k}^{l}|x_{k}^{n},x_{k+1}^{n},Y_{k})$ is normal distribution, the time propagation for the linear state can be performed by applying KF.

(6) $p\left(x_{k+1}^{l}|x_{k}^{n},x_{k+1}^{n},Y_{k}\right)\to p\left(x_{k+1}^{l}|x_{k+1}^{n},Y_{k}\right)$

Marginalization of $p(x_{k+1}^{l}|x_{k}^{n},x_{k+1}^{n}$ for $x_{k}^{n}$ is as follows.

(A12) $$\begin{array}{ccc} p(x_{k+1}^{l}|x_{k+1}^{n},Y_{k}) & = & \int p\left(x_{k+1}^{l}|x_{k}^{n},x_{k+1}^{n},Y_{k}\right)p\left(x_{k}^{n}|x_{k+1}^{n},Y_{k}\right)dx_{k}^{n}\\ & = & \int p\left(x_{k+1}^{l}|x_{k}^{n},x_{k+1}^{n},Y_{k}\right)\frac{p\left(x_{k+1}^{n}|x_{k}^{n},Y_{k}\right)p\left(x_{k}^{n}|Y_{k}\right)}{p(x_{k+1}^{n}|Y_{k})}dx_{k}^{n} \end{array}$$

where $p(x_{k+1}^{n}|x_{k}^{n},Y_{k})$ is the probablility vale at $w^{\prime }=x_{k+1}^{n}-f_{k}^{n}\left(x_{k}^{n}\right)$ for the new process noise $w^{\prime }$ in Step (3). That is, it is $p\left(x_{k+1}^{n}|x_{k}^{n},Y_{k}\right)=p_{w^{\prime }}\left(x_{k+1}^{n}-f_{k}^{n}\left(x_{k}^{n}\right)\right)$, so the above equation can be rewritten as follows.

(A13) $$\begin{array}{c} p\left(x_{k+1}^{l}|x_{k+1}^{n},Y_{k}\right)=\frac{1}{p(x_{k+1}^{n}|Y_{k})}\int p\left(x_{k+1}^{l}|x_{k}^{n},x_{k+1}^{n},Y_{k}\right)p_{w^{\prime }}\left(x_{k+1}^{n}-f_{k}^{n}\left(x_{k}^{n}\right)\right)p\left(x_{k}^{n}|Y_{k}\right)dx_{k}^{n} \end{array}$$
